# Structural-and-dynamical similarity predicts compensatory brain areas driving the post-lesion functional recovery mechanism

**DOI:** 10.1093/texcom/tgad012

**Published:** 2023-07-17

**Authors:** Priyanka Chakraborty, Suman Saha, Gustavo Deco, Arpan Banerjee, Dipanjan Roy

**Affiliations:** Cognitive Brain Dynamics Lab, National Brain Research Centre, NH-8, Manesar, Haryana 122051, India; Cognitive Brain Dynamics Lab, National Brain Research Centre, NH-8, Manesar, Haryana 122051, India; Center for Brain and Cognition, Computational Neuroscience Group, Department of Information and Communication Technologies, Universitat Pompeu Fabra, Barcelona, Spain; Institució Catalana de la Recerca i Estudis Avançats, Barcelona, Spain; Department of Neuropsychology, Max Planck Institute for Human Cognitive and Brain Sciences, Leipzig, Germany; School of Psychological Sciences, Turner Institute for Brain and Mental Health, Monash University, Melbourne, Australia; Cognitive Brain Dynamics Lab, National Brain Research Centre, NH-8, Manesar, Haryana 122051, India; Cognitive Brain Dynamics Lab, National Brain Research Centre, NH-8, Manesar, Haryana 122051, India; School of AIDE, Center for Brain Research and Applications, IIT Jodhpur, NH-62, Surpura Bypass Rd, Karwar, Rajasthan 342030, India

**Keywords:** virtual lesion, structurally similar areas, feedback inhibition control, homeostasis, dynamically similar areas

## Abstract

The focal lesion alters the excitation–inhibition (E–I) balance and healthy functional connectivity patterns, which may recover over time. One possible mechanism for the brain to counter the insult is global reshaping functional connectivity alterations. However, the operational principles by which this can be achieved remain unknown. We propose a novel equivalence principle based on structural and dynamic similarity analysis to predict whether specific compensatory areas initiate lost E–I regulation after lesion. We hypothesize that similar structural areas (SSAs) and dynamically similar areas (DSAs) corresponding to a lesioned site are the crucial dynamical units to restore lost homeostatic balance within the surviving cortical brain regions. SSAs and DSAs are independent measures, one based on structural similarity properties measured by Jaccard Index and the other based on post-lesion recovery time. We unravel the relationship between SSA and DSA by simulating a whole brain mean field model deployed on top of a virtually lesioned structural connectome from human neuroimaging data to characterize global brain dynamics and functional connectivity at the level of individual subjects. Our results suggest that wiring proximity and similarity are the 2 major guiding principles of compensation-related utilization of hemisphere in the post-lesion functional connectivity re-organization process.

## Introduction

One of the fundamental queries in systems neuroscience is ”How does the brain adapt to post-lesion recovery and whether some general normative patterns exist irrespective of individual variations in lesion extent and locations? What are compensatory mechanisms critical to brain network recovery during the post-injury period, including changes in local homeostasis and widespread coordinated cortical activity?” Here, we propose a detailed computational framework using structural-and-functional equivalence principles by demonstrating that the brain’s normative spontaneous dynamical pattern is compensated by restoring local homeostasis post-lesion, and the compensatory brain areas are primarily recruited utilizing 2 major guiding principles related to network topology, structural similarity and wiring proximity of compensation-related utilization of hemisphere (CRUH) in the post-lesion functional re-organization.

The term ”focal lesion” (Gratton et al. 2012; Aerts et al. 2016) refers to biological perturbation to the anatomical architecture, e.g. damage of a region due to stroke (ischemic stroke due to atherosclerosis, hemorrhagic stroke) (Aerts et al. 2016), traumatic brain injury (TBI) (Werner and Engelhard 2007), glioma (Duffau et al. 2003) can qualitatively alter short- and long-term brain functions. In lesions, neurons that are deprived due to lack of oxygen, and energy from standard metabolic substrates, cease to function in seconds and show severe signs of anatomical damage after 2 minutes (Murphy et al. 2008). In the first few days or weeks after injury, regular patterns of synaptic activity in peri-infarct (Gao et al. 1999; Bolay et al. 2002; Carmichael et al. 2004; Brown et al. 2009) and even distant functionality-related structure are interrupted (Bütefisch et al. 2003). Failures in the energy-dependent processes due to loss of inputs from adjacent tissue (Hossmann 2006) lead towards cell death (Besancon 2008), abnormal neuronal firing rates (Maeda and Miyazaki 1998), and may even lead to delayed neuronal injury (Arundine 2003), which inflict local to global level excitation–inhibition (E–I) balance on the neuronal network (Wang 2003; Arundine and Tymianski 2004). These mechanical and cellular alterations can cause chronic functional disabilities, including motor deficits (e.g. hemiparesis), sensory (e.g. hemianopia), and higher-order cognitive processes (e.g. aphasia, hemispatial neglect) (Musuka et al. 2015) and abnormal movement synergies (Willer et al. 1993; Cirstea and Levin 2000).

Studies have revealed the mechanism for lesion recovery and identified associated factors in primate, and non-primate (Kolb and Whishaw 1998; Witte 1998; O’Reilly et al. 2013). For example, the cerebral cortex triggers a plastic mechanism in adjacent and remote areas in post-lesion phases, correlated with limited, spontaneous restoration of function (Gerloff et al. 2006; Nudo 2007; Alia et al. 2017). Two significant factors are involved in the plasticity mechanism in lesion recovery (Murphy and Corbett 2009), (i) an amount of surviving diffuse and redundant connectivity in the central nervous system, and (ii) new functional circuits can form through remapping between related cortical regions. Homeostatic plasticity is a negative feedback-mediated form of plasticity, also known as synaptic scaling (Murphy and Corbett 2009), that keeps network activity at the desired set point (Turrigiano and Nelson 2004). It helps maintain a stable ratio of excitation and inhibition and sustains the desired working point. Nevertheless, local E–I homeostasis engenders functional recovery by increasing excitation and attenuating inhibition in both perilesional and distant cortical areas (Buchkremer-Ratzmann et al. 1996; Bütefisch et al. 2003; Huynh et al. 2016). In addition, enhancement of cortical excitability in surviving cortical areas would compensate for the lost structural circuits (Carmichael and Chesselet 2002) and functional deficits (Nelles et al. 1999; Ward 2005; Winhuisen et al. 2005).

Other key investigations suggest that graph theoretical properties of structural and functional networks plays a crucial role in capturing several aspects of lesion-induced alterations in topological properties of large-scale structural and functional brain networks (Alstott et al. 2009; Crofts et al. 2011; Adhikari et al. 2015; Griffis et al. 2019; Moreira da Silva et al. 2020). However, these studies have yet to systematically investigate region-specific roles in post-lesion functional restoration of brain networks. A recent longitudinal study on mTBI showed notable changes in structural and functional brain networks in the post-lesion recovery phase (Dall’Acqua et al. (2017). However, they did not identify specific regions participating in the functional recovery process. They found no association between damaged functional and structural connections after TBI (Dall’Acqua et al. (2017).

Previous studies have reported network properties, such as nodal strength, participation coefficient, and modularity, played a decisive role in finding the short-term and long-term effects of lesion (Vattikonda et al. 2016; Adhikari et al. 2017; Tao and Rapp 2021). However, the region-specific role of anatomical networks in association with the post-lesion global functional recovery still needs to be fully uncovered and remains an open question. From a dynamical systems perspective, the brain is a spatially organized system (Lashley 1950) with time-dependent signal propagation along multiple pathways, each capable of adapting to changes in transmission fidelity (Murphy and Corbett 2009). Thus, brain dynamics is governed by underlying anatomy, and the underlying intrinsic biological parameters (Caeyenberghs et al. 2013). However, regional specificity in association with the intrinsic parametric role must be elucidated as to how specific regions may play a vital role by adapting intrinsic parameters in shaping emerged brain dynamics to compensate for structural damage following lesions. With this knowledge gap and motivation, we hypothesize that regions with similar incoming and outgoing connections corresponding to a lesion site, labeled as similar structural areas (SSAs) in this study, could be the potential candidate for re-establishing E–I balance (at the level of both local and global brain scale) in the post-injury period. Hence, the prediction of SSAs is one of the fundamental contributions of this study. The second fundamental contribution to identifying dynamically similar areas (DSAs) using readjustment time, a dynamic measure indicating the re-establishment of local E–I balance post-lesion. For example, a region that helps compensate for motor deficit should get incoming motor information from its adjacent or distant areas, thus would be functionally relevant and structurally equivalent. SSA may form complementary or redundant connections in the surviving areas, providing an alternative pathway for information fidelity after the lesion. Thus, SSAs (or DSAs) could lead the adaptive mechanism to compensate for lost local homeostasis and inter-areal excitability, further reshaping collective activity. It can be noted that SSAs are identified from a healthy brain connectome, which captures the structural features of an individual. DSAs are predicted based on the structural connectivity of the lesion node’s neighbors and collective population dynamics of the remaining anatomical network (excluding the lesion site). Thus, the prediction of DSAs relies on the interplay between the structural features and underlying model parameters. It is also important to note that brain damage (TBI, stroke, epilepsy) causes loss of structural information. Hence, identifying compensatory brain regions (SSAs) from the lesion brain is no longer available. However, using DSAs, we can identify these potential compensatory areas corresponding to a specific lesion site. These compensatory areas may drive functional compensation and post-lesion functional recovery. Thus, the third contribution of the study is that DSA offers a methodological improvement over the traditional method of relying on healthy structural connectivity features (e.g. the Jaccard coefficient). Finally, the study provides a fresh perspective on how certain notable areas (ipsilesional and contralesional) influence post-lesion recovery mechanisms, specifically, compensation-related utilization of hemispheres.

We have systematically addressed the following questions: (i) What changes in E–I balance cause altered neural activity after early brain injury due to anatomical network damage? (ii) Which are the notable areas that re-adjust their inhibitory weights to balance E–I homeostasis and sustain a target firing rate $\sim 4$ Hz? (iii) What processes are related to the post-injury functional re-organization within the surviving structural network? To address these questions, we utilized 2 measures, e.g. time to re-establish E–I balance in local areas and modulated local inhibitory weights. Next, we find signatures from the structural properties in correlation with coordinated neural dynamics, and finally, identify the mechanisms displaying correlations between the parameters controlling local E–I homeostasis and structural network similarity measure, e.g. the Jaccard coefficient. We provide demonstrative evidence that we can predict brain areas initiating compensatory post-lesion adaptive mechanisms using in silico stimulation of the personalized whole-brain mean field model. As a proof-of-concept, we use the structural similarity and functional E–I homeostatic mechanisms as equivalence principles to demonstrate that it can find accurate ways to pinpoint a general road map to functional brain network recovery overcoming lesion diversity.

## Material and methods

### Participants

Resting state MRI data from 49 healthy subjects (31 females), ages ranging from 18 to 80 years (mean age 41.55 $\pm$ 18.44 years), have been collected at Berlin Center for Advanced Imaging, Charité University Medicine, Berlin, Germany (Schirner et al. 2015). The participants are healthy, and no history of neurologic or psychiatric conditions was reported in Schirner et al. (2015). All participants gave written informed consent to the group (Schirner et al. 2015), and the study was performed under the compliance of laws and guidelines approved by the ethics committee of Charité University, Berlin, Germany.

### Anatomical connectivity

Resting-state MRI, diffusion-weighted MRI, and functional MRI were performed on a 3 Tesla Siemens Tim Trio MR scanner using a 12-channel Siemens head coil. Detailed information on the data acquisition parameters can be found in Schirner et al. (2015). We did not process the raw data. The data were pre-processed, and structural connectome was generated previously, using the pipeline by Schirner et al. (2015).The cortical gray matter was parcellated into 34 regions of interest (ROIs) in each hemisphere following the Desikan-Killiany parcellation (Desikan et al. (2006). The ROIs with their abbreviations are listed in Table S1 of the Supplemental Material.

### Empirical functional connectivity

Participants are subjected to a functional MRI scan in eyes-closed awake resting-state condition. Resting-state BOLD activity was recorded for a duration of 22 minutes (TR = 2 s) (Schirner et al. 2015). The pre-processing steps are detailed in the Supplemental Material. After pre-processing, the BOLD time series for each region was aggregated and z-transformed. The pairwise Pearson correlation coefficient was computed to obtain the resting-state functional connectivity (rsFC) matrix for each subject.

### Definitions and descriptions

Before outlining the pipeline and workflow, it is important to define and describe the terminologies relevant to the study. The following terms are defined below: Jaccard coefficient, virtual lesion, DMF model, virtual lesion model, re-adjusted inhibitory weights, and time to reach E–I balance.

**Jaccard coefficient ($JC$)**: The $JC$ is used to measure the pairwise correlation between any 2 brain areas. It is defined as the ratio between the sum of the weights of their common neighbors and the total weights of their neighbors (Hilgetag et al. 2000). The weighted Jaccard coefficient is expressed as $JC=\frac{A \cap B} { A \cup B}$, where $A$ and $B$ represent the neighbors (specifically, the edge strengths with neighbors) of any 2 brain areas from the anatomical connectome of healthy subjects. The JC is measured in the healthy brain before the occurrence of a virtual lesion at the level of an individual subject.

**Similar structural areas (SSAs)**: $JC$ of a lesion node is sorted in descending order and the top 25% areas having higher $JC$ values are referred to as SSAs. SSAs correspond to a lesion site share a similar topological property with the selected lesion node.

**Whole-brain computational model**: We use a reduced dynamic mean field (DMF) model (Wong and Wang 2006) to engender lesion effects. The DMF is an approximation of a spiking network model (Deco and Jirsa 2012; Deco et al. 2014) consisting of populations of excitatory and inhibitory neurons with excitatory NMDA synapses and inhibitory GABA synapses. DMF is described by a set of coupled nonlinear stochastic differential equations given below, 


(1) \begin{align*} &I_{i}^{(E)}=w_{E}I_{0}+w_{+}J_{N}S_{i}^{(E)}+GJ_{N}\sum_{j=1}^{N}C_{ij}S_{j}^{(E)}-J_{i}S_{i}^{(I)}, \nonumber \\ &I_{i}^{(I)}=w_{I}I_{0}+J_{N}S_{i}^{(E)}-w_{II}S_{i}^{(I)}, \nonumber \\ &r_{i}^{(E)}=\frac{a_{E} I_{i}^{(E)}-b_{E}}{1-e^{-d_{E}\left( a_{E} I_{i}^{(E)}-b_{E} \right)}}, \quad r_{i}^{(I)}=\frac{a_{I} I_{i}^{(I)}-b_{I}}{1-e^{-d_{I}\left( a_{I} I_{i}^{(I)}-b_{I} \right)}}, \nonumber\\ &\frac{dS_{i}^{(E)}}{dt}= -\frac{S_{i}^{(E)}}{\tau_{E}}+\left(1-S_{i}^{(E)}\right)\gamma r_{i}^{(E)}+\sigma\nu_{i}(t), \nonumber\\ &\frac{dS_{i}^{(I)}}{dt}= -\frac{S_{i}^{(I)}}{\tau_{I}}+r_{i}^{(I)}+\sigma\nu_{i}(t). \end{align*}

$I_{i}^{E,I}$ is the input current to area $i$ and superscripts represent excitatory ($E$) and inhibitory($I$) populations in that area. $r_{i}^{E,I}$ is the population firing rate of excitatory or inhibitory populations of area $i$. $S_{i}^{E, I}$ is the average excitatory (inhibitory) synaptic gating variable of area $i$. $I_{0}$ is the effective external input scaled by $w_{E}$ and $w_{I}$ for excitatory and inhibitory populations. $w_{+}$ is the local excitatory recurrence, $J_{N}$ is the excitatory synaptic coupling, and $J_{i}$ is the local feedback inhibitory synaptic coupling. $w_{II}$ is the local inhibitory recurrence. $C_{ij}$ is the $i,j^{th}$ entry in the SC matrix, obtained from diffusion imaging (MRI data), that scales the long-range excitatory currents between $j^{th}$ and $i^{th}$ regions. $G$ represents global coupling strength which scales long-range excitatory connections. To find optimal $G$, the DMF model is simulated for different values of $G$. The optimal value of the coupling strength is optimized by fitting the algorithm on the Berlin dataset by Vattikonda et al. (2016). For detailed descriptions, check Fig. 3a in Ref. Vattikonda et al. (2016). It was assumed that the DMF exhibited neural complexity following a balanced homeostasis resembling the resting state at this optimal parameters set. The optimal value of $G$ is chosen based on the highest correlation between empirical and simulated FC when the excitatory firing rate sustains at $\sim$4 Hz within all brain regions (Burns and Webb 1976; Shadlen and Newsome 1998; Deco et al. 2008; 2014; Adhikari et al. 2015). Firing rates are statistical measures that describe the average activity of neurons over a specific time period. Mean spontaneous rates of 4.5 Hz and stimulus-evoked rates of 10.6 Hz were observed in broad-spiking neurons (Mitchell et al. 2007; Deco et al. 2008; 2014; Haider et al. 2013). MATLAB 2021b is used to perform all the numerical simulations. Euler’s method, with a step size of 1 ms, has been used to generate the synaptic activity of each area. The whole model is simulated for 10 minutes, where the first 2 minutes of transients are discarded. Default parameter values are selected from Ref. Deco et al. (2014) and presented in Supplemental Material, Table S2.

**Feedback inhibition control**: FIC algorithm, proposed by Deco et al. (2014) is a recursive process to establish and maintain E–I balance in individuals and across all cortical subunits. The detailed FIC steps are found in the supplementary material. We simulated the model with the FIC algorithm for 10 $s$ time windows.

**Virtual lesion**: The virtual focal lesion is introduced into an individual subject’s SC by targeted node removal. Expressly, all the connections to and from the focal lesioned site have been set to zero in the SC matrix.

**Virtual lesion model**: We put the DMF model on top of a virtually lesioned SC of a single subject, together labeled as a virtual lesion model. Individual node dynamics are governed by the stochastic DMF model spatially coupled via lesioned SC matrix. In principle, any other form of lesioned SC (real, virtual), if incorporated into the dynamical model, can produce similar effects of lesion depending on the lesion type, location, and extent.

**Post-lesion modulated local inhibitory weight ($J^{\prime }$)**: The modulated local inhibitory synaptic weights ($J_{i}^{\prime }$) at individual nodes, in case of a lesion are compared against the weights ($J_{i}$), estimated from healthy brain condition. The change in inhibitory synaptic weight for $i^{th}$ area is calculated as: $dJJ_{i}$ = $J_{i}^{\prime }$-$J_{i}$.

**Re-adjustment time ($RT$):** In the process of FIC, different brain regions require different simulation times to achieve the optimum firing rate and a balanced E–I homeostasis. While resetting the E–I balance until the entire brain reaches the desired balanced state, we track the simulated time windows of individual regions, defined as the re-adjustment time ($RT$). We store the elapsed time $RT$ and $J^{\prime }$ for all regions during the FIC process, where the measuring units are $s$ and $mMol$, respectively. 
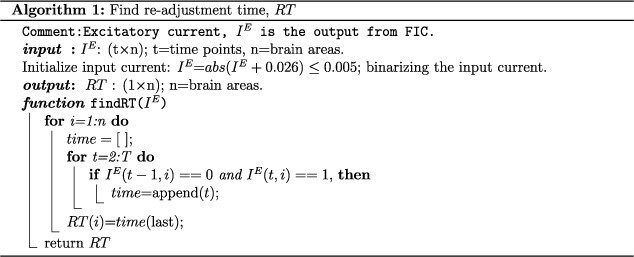


**Dynamical similar areas (DSAs):** First, we sort both $RT$ and $|dJJ|$ in descending order. We choose the top 25% areas from the sorted $RT$ and $dJJ$. We then find the intersection of the sorted areas to identify the common areas in $RT$ and $dJJ$. The identified common areas from the 2 measures are the DSAs. 


\begin{align*} &DSA= AI_{RT}|_{1:N} \cap AI_{dJJ}|_{1:N},\end{align*}

where $AI_{RT}$ and $AI_{dJJ}$ represent the area indices identified from the sorted $RT$ and $|dJJ|$ respectively. $N$= total number of ROIs $\times$ 25%.

**Overlapping between SSAs and DSAs:** We consider only those regions which are common in both SSAs and DSAs, i.e. the overlapping regions between SSAs and DSAs.

### Summary of the method

We used a personalized mean-field model trained by healthy SC features and FC as inputs where the system parameters were optimized based on FC–FC fitting (Deco et al. 2014; Vattikonda et al. 2016). Once optimized, the model could learn healthy connectome from non-invasive MRI and fMRI data and map inter-individual functional differences. Later, we used this model and fed the damaged SC (virtually lesioned connectome) to generate subject-wise FCs for 2 lesion conditions. Hence, only symmetry breaking in the system is induced by individuals’ anatomical topology. This primarily motivates us to examine the inter-subject variability and consequences of different lesion locations based on the topological differences. The symmetry-breaking condition is utilized to extract subject-wise differentiating features from anatomical connectome.

## Results

**Fig. 1:**
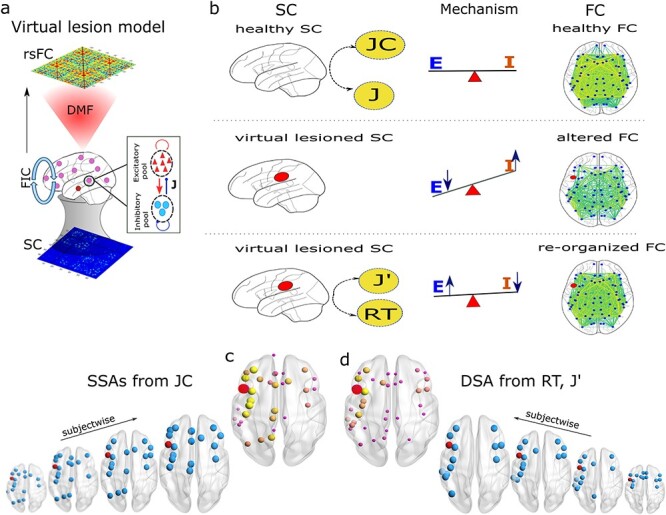
Workflow. (a) Schematic of the virtual lesion model. SC is generated based on the Desikan-Killiany atlas with 68 regions of interest (ROI). A dynamical mean field model (DMF) is spatially connected via virtually lesioned SC. The DMF model generates resting-state neural activity using a feedback inhibition control (FIC) algorithm. Synthetic BOLD series are generated from the neural signals using a hemodynamic model. The FIC is a recursive process of adjusting local inhibitory feedback weight ($J$). At optimal conditions, each region maintains balanced homeostasis and an excitatory firing rate between $2.63$-$3.55$Hz. The pairwise Pearson correlation coefficient determines the simulated resting-state FC. (b) Our analysis is performed considering 3 conditions, placed in 3 rows. The top row shows healthy conditions. First, the Jaccard coefficient ($JC$) matrix is calculated from a healthy SC. Next, the DMF model coupled via healthy SC has been run with the FIC algorithm establishing E–I balance. The final value of each brain region’s feedback synaptic inhibitory weights ($J$) is stored beside the synaptic activity. The synaptic gating variables are further processed to generate model-based rsFC. The seesaw represents the status of the global E–I balance state. The obtained $J$ has been used further as initial values for inhibitory coupling weights in the model simulation for the rest of the 2 conditions. In the second row, a virtual lesion is introduced to the SC by setting all rows and columns equal to zero. Next, the virtual lesion model is run without the FIC to generate model-based altered FC. The seesaw represents an imbalanced E–I state as an early impact of the lesion. The lower row depicts the third condition when E–I balance is restored in the brain. The virtual lesion model is simulated with the FIC algorithm. Model parameters, such as modulated local synaptic inhibitory weights ($J^{\prime }$) and re-adjustment time ($RT$), are stored for further analysis. Yellow circles show the model parameters of our interest. Two representative results are shown in (c) and (d). (c) Identified SSAs are shown for individual subjects and group levels. (d) Estimated DSAs using $RT$ and $J^{\prime}$ are shown.

To test our hypothesis, we simulate a virtual lesion model. The virtual lesion is introduced by deleting incoming and outgoing connections of a node in the structural connectome of healthy subjects (Alstott et al. 2009; Vattikonda et al. 2016). Fig. 1a shows a large-scale dynamical mean field (DMF) model (Cabral et al. 2012; Deco et al. 2014) on top of the virtually lesioned structural connectivity (SC), labeled as the virtual lesion model. A feedback inhibition control (FIC) algorithm (Deco et al. 2014), a negative feedback-mediated form of plasticity, re-adjusts local inhibition synaptic weights to restore E–I balance and target firing rate of $\sim$4 Hz (Burns and Webb 1976) in the post-injury period. We did not consider other virtual lesion types, such as edge deletion or multi-region damage (Alstott et al. 2009; Vattikonda et al. 2016). The pre-processed data of 49 healthy subjects are taken from the Berlin data set (Schirner et al. 2015). Desikan-Killiany (Desikan et al. 2006) parcellation divides the brain into 68 regions of interest.

The mathematical framework is set for 3 conditions based on the SC status and the E–I balance state. Three rows display the 3 operant conditions for model simulation in Fig. 1b.

The top row in Fig. 1b describes the healthy condition when SC remains intact, and the E–I balance is maintained. We derive the JC matrix from the healthy SC. The SSAs correspond to a given lesion site from a healthy individual’s SC based on the JC measure before lesion occurrence. We store the local inhibitory weights ($J_{i}$) in parallel by running the FIC algorithm. The FIC algorithm helps in establishing E–I balance in the whole brain. Under the same operant condition, we synthetically generate healthy FC from the model simulation. The obtained inhibitory synaptic weights ($J_{i}$) are further considered as initial values for inhibitory plasticity in the following 2 conditions for the virtual lesion analysis.

In the middle row of Fig. 1b, the virtual lesion is introduced into the healthy SC, resulting in loss of E–I balance, i.e. a short-term loss of E–I balance due to lesion impact, as our second condition. Next, we simulate the virtual lesion model without FIC to capture altered FC for further comparison with healthy and other post-lesion conditions.

The lower row in Fig. 1b depicts the condition after the lesion when the FIC restores the E–I balance. At this condition, the adaptive nature of the brain re-adjusts the local inhibitory weights to restore the desired E–I balance. It allows the damaged brain to sustain at desired firing rate $\sim$4 Hz. The virtual lesion model with the FIC is simulated to generate re-organized FC, which is compared against the altered FC obtained from the previous condition. While we numerically simulate the lesioned model, we store re-adjusted synaptic inhibitory weights ($J^{\prime }$) and capture re-adjustment time ($RT$) during the re-establishment of local E–I balance in an individual area. Further, the curated $RT$ and $J^{\prime }$ are correlated with the measured JC values corresponding to a lesion site. As we are more interested in finding changes in inhibitory synaptic weights, we calculate $dJJ_{i}$= $J_{i}^{\prime }$-$J_{i}$ for all areas. Based on the 2 measured quantities ($RT$ and $J^{\prime }$), we estimate DSAs while observing the global homeostatic condition. We unveil the functional affinity for alternation and re-organization pattern of the brain after lesion by correlating SSAs, identified from anatomical measure $JC$, and DSAs from simulation parameters ($RT$, $dJJ$).

It is worth mentioning that the first condition is a one-time process, whereas the following 2 steps are repeated for different virtual lesion sites at the single subject level. All 3 steps have been repeated for individual subjects and further analyzed at the group level.

We have performed 2 levels of analysis, (i) anatomical level analysis and (ii) functional alteration/re-organization analysis. Significant changes between healthy and altered FCs and altered and re-organized FCs are captured by parametric test, an independent t-test analysis. Functional network properties (measuring network resilience, segregation, and integration) such as modularity, transitivity, global efficiency, and average characteristic path length are derived to evaluate functional alteration due to short-term loss of E–I balance (mimicking early lesion phase) and long-term functional re-organization in the post-lesion phase.

### Structural connectivity analysis

#### Identify SSAs using Jaccard coefficient

**Fig. 2:**
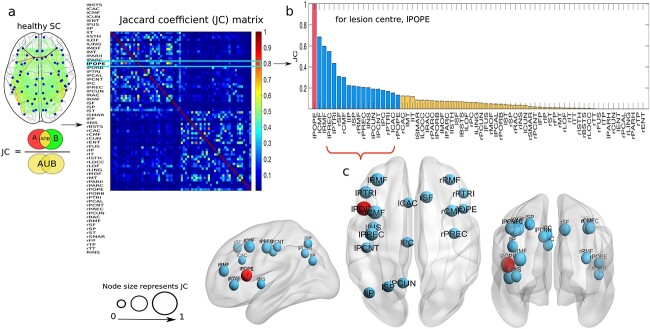
**Similar structural areas (SSAs) identified by $JC$ for a single subject.** (a) $JC$ is evaluated from healthy SC, where $A$ and $B$ are any 2 regions of interest. (b) Distribution of $JC$ values, considering the lesion to be at left pars opercularies (lPOPE), are plotted in descending order. The red bar is the selected lesion site (lPOPE). The top 25% areas from the $JC$ values are considered SSAs, shown in blue. The rest of the lower similar areas are shown in yellow bars. (c) Identified SSAs are shown in sagittal, axial, and coronal brain views. Node sizes represent $JC$ values.

Weighted $JC$ are calculated from the individual subject’s healthy SC. High $JC$ values imply a high structural similarity, whereas low values yield lesser similarity corresponding to an area of interest (could be a lesion center). A threshold is put on the obtained JC values corresponding to a lesion site to identify higher similar areas. The top 25% areas with higher $JC$ values are considered as SSAs. Figure 2a shows $JC$ matrix obtained from a healthy SC (without lesion). Descending order distribution of $JC$ values corresponding to the lesion at lPOPE is plotted in Fig. 2b. The top 25% similar areas are shown in blue bars and the rest in yellow. In Fig. 2c, only the top 25% similar areas (blue nodes) are plotted on the brain surface using BrianNet viewer. Node size implies $JC$ values. The top 25% SSAs of lPOPE are lCMF, lRMF, lPTRI, lPREC, rSF, rCMF, lIP, lSP, rRMF, rPREC, lINS, lPCUN, lPCNT, rPTRI, rPOPE, written in descending order from the similarity indices. It is observed that the higher similar areas, e.g. rostral, caudate middle frontal cortex, parietal cortex, insular cortex, and primary and supplementary motor cortex, are found both in ipsilesional and contralesional hemispheres. SSAs corresponding to lPOPE for different subjects are shown in Supplemental Material, Figure S1a. The SSAs for other lesioned sites are shown in Supplemental Material. Right POPE has SSAs such as lCMF, lPTRI, lPOPE, and lRMF in homotopic regions to the left hemisphere (Supplemental Material, Fig. S1c), including rostral, caudal, precentral, and postcentral gyrus. SSAs of primary motor regions (left precentral gyrus, lPREC) are distributed in both hemispheres, including the caudal (l/rCMF), rostral (l/rRMF), frontal (l/rSF) cortex. Regions are also in the parietal (lIP, lSP, lPCUN) and insular (lINS) cortex for the left hemisphere (Supplemental Material, Fig. S1e). The left lateral occipital (lLOCC), part of the visual cortex has SSAs mainly in the ipsilesional hemisphere ranging from the parietal lobe (lIP, lPCAL, lSP, lSMAR, lISTH) to the middle frontal lobe (lCMF) via temporal regions (lST, lMT, lTT, lFUS) and insular (lINS) cortex (Supplemental Material, Fig. S1g). Other structural similar areas for different regions are tabulated in Supplemental Material, Table S3.

#### Correlation analysis between anatomical ($JC$) and dynamical ($RT$, $dJJ$) measures

**Fig. 3:**
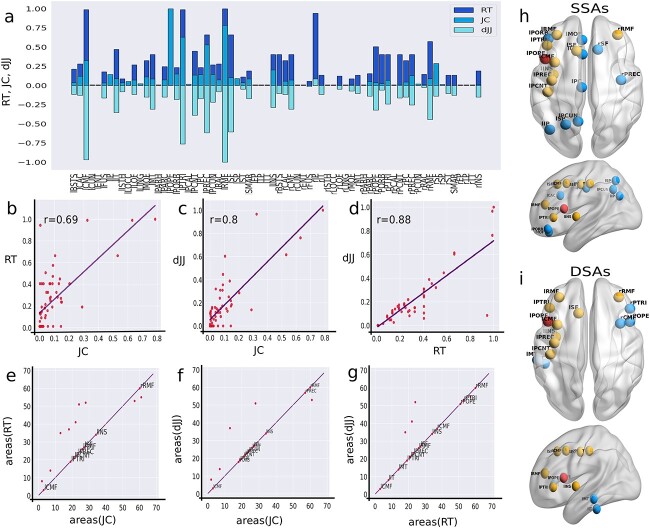
**Correlation between anatomical and dynamical parameters corresponding to the lesion at lPOPE.** (a) Area-wise distribution of $JC$, $RT$, and $dJJ$ for a single subject. From the derived $JC$ matrix on healthy SC, we only choose the $JC$ values considering the lesion center to be at lPOPE. Next, we take absolute $dJJ$ values for the rest of our analysis. (b-d) Scatter plots show the correlation between $JC$-$RT$, $JC$-$dJJ$, and $RT$-$dJJ$, respectively. Violet lines denote a positive correlation between any 2 given measures. Correlation values ($r$) are given at the top of each plot. (e-g) Areas of interest are identified considering a higher correlation between $JC$-$RT$, $JC$-$dJJ$, and $RT$-$dJJ$. The regions on the purple lines are common within the 2 measures. We choose DSAs from the overlapped areas in both $RT$ and $dJJ$, i.e. the areas lie on the purple line in (g). (h) To compare between SSAs and DSAs, we repeat the identified SSAs here. (i) The estimated DSAs are shown in sagittal and coronal brain view. The red sphere is the lesion center, lPOPE. Common regions from SSAs and DSAs are shown in yellow and non-overlapping in blue.

The $JC$ is derived from a healthy SC and used to test our hypothesis, whether the SSAs corresponding to a lesion site are essential in restoring E–I homeostasis in local regions and eventually within whole cortical systems. Model-based measurable parameters, re-adjustment time ( $RT$), and change in inhibitory weights ($dJJ$) have been used to predict the dynamically similar areas (DSAs). We have simulated the virtual lesion model when the DMF model is spatially connected via the virtually lesioned SC of a single subject. Area-wise distribution of $JC$, $RT$, and $dJJ$ correspond to the lesion site at lPOPE is shown in Fig. 3a. A higher $RT$ node value implies a more extended time required to reach the desired threshold in excitatory synaptic current for balancing the E–I ratio. A negative value of $dJJ$ for an area implies a reduction in its inhibitory weight, i.e. a decrease in inhibition of that area. We take absolute $dJJ$ for better visualization and description in our analysis. We find a positive association ($r=0.69$) between $JC$ and $RT$, which yields that SSAs take longer to re-adjust the E–I balance, as shown in Fig. 3b. Conversely, a positive correlation between $JC$ and $dJJ$, fitted by a linear fitting model with $r=0.8$ (see Fig. 3c). It indicates that the SSAs have tuned their local inhibitory weights. We find a positive correlation between $RT$ and $dJJ$, fitted by a linear regression model ($r=0.88$; see Fig. 3d). Overall observations suggest that SSAs have a strong correlation with DSAs, which implies the SSAs take longer to modulate their inhibitory weights to settle the neural activity at the desired set point, i.e. balanced E–I ratio and target firing rate of $\sim$4 Hz.

Next, we aim to estimate the areas with a higher positive association measured from the correlation between $JC$ and $RT$, or $JC$ and $dJJ$. We have selected the regions that lie on the diagonal line (violet line) only, shown in Fig. 3e, which are common in both independent measures. The estimated regions, such as lCMF, lPTRI, lPCNT, lPREC, lRMF, lSF, lINS, and rRMF, are common in both SSAs and DSAs, with larger $JC$ and $RT$ values. Similarly, we identify regions from the correlations between $JC$ and $dJJ$, such as lCMF, lPORB, lPTRI, lPCNT, lPREC, lPCUN, lRMF, lSF, lINS, rPREC, and rRMF, shown in Fig. 3f. Subsequently, we identify the predicted brain areas obtained from the substantial overlap between their $RT$ and $dJJ$ values. This approach identifies the following brain regions lCMF, lIT, lMT, lPTRI, lPCNT, lPREC, lRMF, lSF, lINS, rCMF, rPOPE, rPTRI, and rRMF (see Fig. 3g). The SSAs, selected from anatomical measure ($JC$), are shown in Fig. 3h. The DSAs, identified from dynamical measures ($RT$ and $dJJ$), are plotted in Fig. 3i on the glass brain in sagittal and axial view. Yellow nodes in Fig. 3h and i are common in both SSAs and DSAs, where blue ones are the non-overlapping areas. The left pars opercularis (lPOPE) lesion site is shown in the red sphere. Interestingly, areas identified by the 2 independent analyses, i.e. SSAs and DSAs, have more than 60% overlapped regions corresponding to the lesion site, lPOPE. The estimated DSAs corresponding to lesion centers at lPOPE, rPOPE, lPREC, and lLOCC in different subjects are shown in Supplemental Material, Figure S1b, S1d, S1f, and S1h, respectively. Other DSAs for different lesion centers are tabulated in Supplemental Material, Table S3. A strong correlation between $JC$ and $RT$ or $JC$ and $dJJ$ and high overlapping between SSAs and DSAs suggest that the predicted areas play a crucial role in re-establishing local E–I balance by calibrating their inhibitory weights and help sustain the target firing rate ($\ 4$ Hz) after the lesion occurrence. Further, we sequentially introduce virtual lesions to all 68 areas. We investigate correlations between $JC$ and dynamical measures ($RT,dJJ$) at the level of single subjects, as depicted in Supplemental Material, Figure S2. The correlation between $JC$ and $RT$ in Figure S2a, and $JC$ and $dJJ$ in Figure S2b is positive for different lesion centers. Except for a few regions, such as lENT, rENT, lPARH, rPARH, lFP, rFP, lTT, rTT, lTP, and rTP, other regions display largely weaker or negative correlations. The estimated SSAs and DSAs are displayed in Supplemental Material, Figure S3a and b. These 10 nodes have less number of connections and nodal strength. Lower strength and degree of a node could be why their SSAs are not participating in E–I balance (Supplemental Material, Fig. S3e and f).

#### Inter-subject and inter-hemispheric variability/similarity

**Fig. 4:**
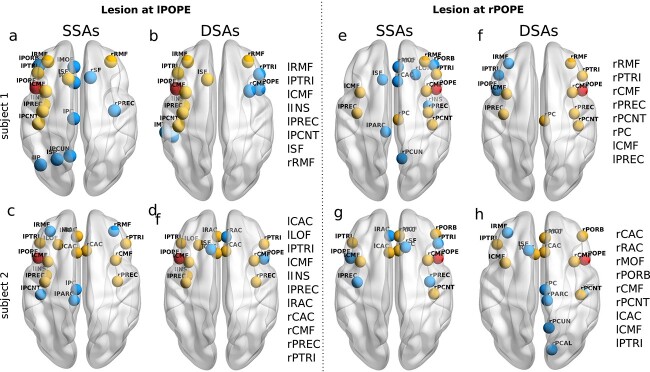
**Inter-subject and inter-hemispheric variability/similarity in SSAs and DSAs.** SSAs corresponding to lesion centers at left and right POPE ( red spheres) are shown respectively in (a, c) and (e, g) for 2 subjects. DSAs are shown respectively in (b,d) and (f,h) for those 2 subjects. Common regions from SSAs and DSAs are shown in yellow spheres. Abbreviations are written on each subplot’s left side. Overlapping areas in SSAs and DSAs are yellow, and non-overlapping are blue.

Fig. 4 shows the results for 2 subjects and lesion sites at 2 hemispheres. We describe the findings from the 2 independent analyses. The SSAs and DSAs corresponding to the lesion site lPOPE are repeated for one subject in Fig. 4a and b. Estimated common areas from both SSAs and DSAs are shown in yellow and non-overlapping in blue. The overlapping regions are lRMF, lPTRI, lCMF, lINS, lPREC, lPCNT, lSF, and rRMF, respectively.

Inter-subject variability is depicted for another subject in Fig. 4c and d. The overlapping regions for this subject are lCAC, lLOF, lPTRI, lCMF, lINS, lPREC, lRAC, rCAC, rCMF, rPREC, rPTRI, displayed in yellow in Fig. 4c and d. Although the lesion centers are similar for both subjects; still, the identified SSAs and DSAs are different in the 2 subjects; compare Fig. 4a and c, or Fig. 4b and d. Variability in SSAs arises from individual subjects’ structural/anatomical differences in brain connectivity and manifest individual-specific SC-FC correlations.

However, it is interesting to note that the areas predicted for lesion recovery by $JC$ are similar to the regions predicted by the dynamical measures ($RT$-$dJJ$) in individual subjects (see Fig. 4a and b or Fig. 4c and d), despite inter-subject structural differences. While comparing the 2 Fig. 4b and d, the estimated DSAs lie in the anterior cingulate cortex (l/rRAC, l/rCAC) for subject-2 (Fig. 4d), whereas no nodes from the anterior cingulate cortex are found for subject-1 (Fig. 4b). Patterns of subject-dependent variability/similarity in the estimated DSAs and SSAs are consistent for different lesion locations and tested for several subjects (Supplemental Material Fig. S1).

Further, we tested our hypothesis for another lesion site in the right hemisphere, say right pars opercularis (rPOPE), for the 2 subjects. We find consistent variability patterns in the results, see Fig. 4e and g or Fig. 4f and h, and similarity in identified regions, comparison between the SSAs and DSAs in Fig. 4e and f or Fig. 4g and h. The overlapped regions from the SSAs and DSAs are shown in yellow, and the non-overlapping in blue.

#### Group-level analysis on SSAs and DSAs

A group-level analysis is performed over all 49 subjects to find the probability of the appearance of a predicted SSA or DSA. We determine the probability of appearance (PA) of an area as a ratio between the number of an SSA (or DSA) that appeared within all the subjects and the total number of subjects as, 


\begin{align*} &PA=\frac{number\ of \ appearance \ of \ an \ area\ in\ all\ subjects}{total\ subject}.\end{align*}

**Fig. 5:**
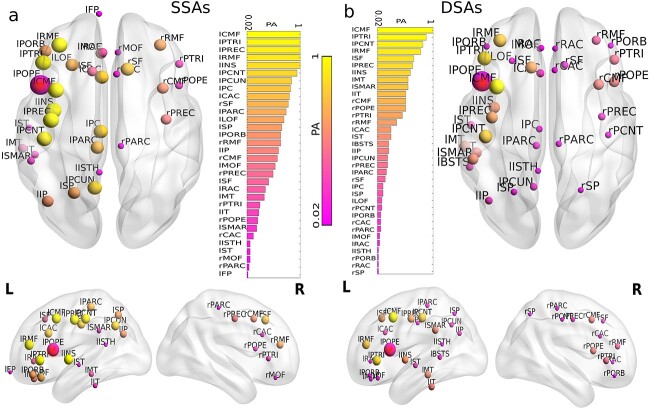
**Group-level analysis on SSAs and DSAs corresponding to lPOPE.** Probability of appearance (PA) of nodes identified by structural and dynamical measures over subjects corresponding to the lesion at lPOPE are illustrated. (a) Axial, sagittal (left and right) view of the brain for SSAs over all subjects and the distribution of PA in descending order for SSAs. (b) Descending order distribution of PA for DSAs, axial and sagittal (left and right) view of the brain for DSAs over all subjects. Yellow areas have higher PA yielding, appearing as SSA and DSA over all subjects. The pink areas appeared less in both SSAs and DSAs. The red node is the lesion site, lPOPE.

The value $PA$=1 corresponding to an SSA (or DSA) implies that it appeared in all the subjects. The values and distribution of the PA for SSAs corresponding to lPOPE are shown in Figs. 5a, and 5b, respectively, and the PA values for DSAs are plotted in Fig. 5c and d. Yellow nodes and bars stand for higher PA values, and pink shade implies lesser PA values, indicated by the color bar. It is observed that the SSAs corresponding to the lesion center at lPOPE, such as lCMF, lPTRI, lPREC, lRMF, and lINS, are found in all 49 subjects (see Fig. 5a and b). Other SSAs, including the postcentral gyrus, precuneus, posterior cingulate, and contra lesional frontal regions, are found in more than 90% of the subjects. SSAs in the right hemisphere, e.g. rPOPE and rPTRI, are found in more than 50% of subjects. Similarly, the DSAs, including lCMF, lPTRI, lPCNT, and lRMF, are found in almost all the subjects. We test the consistency and robustness of our results for other lesion locations; please check Supplemental Material Figure S3a and b, and Figure S3e and f.

Although the identification of SSA based on anatomical property is entirely independent of the estimation of DSA, both these proposed methods can crucially identify and predict similar compensatory candidate brain regions likely initiating the post-lesion recovery process. The higher similarity between SSAs and DSAs signifies that the similar structured areas corresponding to a possible lesion site have modulated their local inhibitory weights and participated in restoring local and global homeostatic E/I balance.

### Functional alteration and re-organization after lesion

We have used statistical tools to investigate how anatomical perturbation to a node affects the global functional organization. The impact of lesion on FC has been categorized into 2 parts: (i) alteration and (ii) re-organization. Simulated FC is obtained from the spatiotemporal BOLD signals using pairwise Pearson correlation. Considering 3 conditions for each subject, we have synthetically generated healthy FCs, altered FCs when E–I balance is lost and re-organized FCs when the E–I balance is restored. Statistical comparison between any 2 conditions, e.g. healthy vs. altered FCs, and altered vs. re-organized FCs, respectively, determines significant differences between generated FCs. First, we calculate the z-score of individual FCs for the 3 conditions. Next, we perform paired sample t-tests to designate the significant global changes in the healthy normative pattern, i.e. deviation from the healthy FC into the altered FC that depicts the direct impact of lesion on collective dynamics. Similarly, after the global restoration of E–I homeostatic balance, the post-lesion functional reconfiguration pattern is investigated by comparing the altered and re-organized FC. The ROI-wise paired t-test is performed for each element in the FC matrix between 2 conditions, e.g. healthy and altered FC group and altered and re-organized FCs, for all subjects. False discovery rate (FDR) is corrected over the obtained $pP$-values from the t-test.

**Fig. 6:**
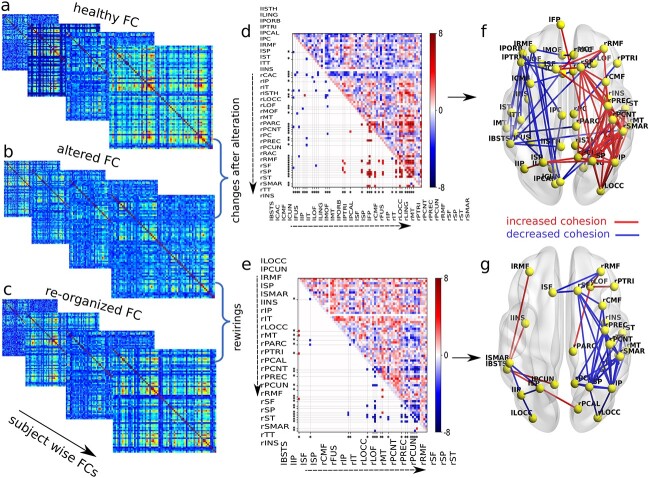
**ROI-wise FC analysis.** (a-c) Model-generated healthy, altered, and re-organized FCs are placed in 3 rows. The virtual lesion is introduced at lPOPE. ROI-wise changes and rewiring between 2 conditions over the subjects are measured using paired t-tests. ROI-wise $t$-statistics are displayed in the upper triangular matrices, comparing healthy and altered FCs in (d) and altered and re-organized FCs in (e). Lower triangular entries are the significant $t$-statistic, putting a threshold on the $p$-values ($p<0.05$, FDR corrected). The color bar indicates the $t$-stat value. Stars at the bottom and left sides of the matrices are significantly changed regions. Glass brain plots in (f) and (g) show, respectively, the significantly changed and rewired links with related regions. Red and blue edges represent the significant increase and decrease in $t$-stat values. (f) Due to lost homeostatic balance, cortical cohesion is reduced in ipsilesional and increased in contralesional. In contrast, (g) spatiotemporal correlation is enhanced in the ipsilesional and attenuated in the contralesional hemisphere after restoring the E–I balance.

Figure 6 shows ROI-wise FC analysis between 2 conditions, e.g. healthy-altered FC and altered-reorganized FC, considering lesion center lPOPE. Subject-wise model-generated healthy FC, altered FC, and re-organized FC are shown in Fig. 6(a-c), respectively. ROI-wise $t$-statistic to find significant changes in the weights between healthy and altered, as well as altered and re-organized FCs, are shown in Fig. 6d and e, respectively. Upper triangular elements in Fig. 6d and e represent the $t$-statistics corresponding to changed weights between any given pair of regions. Lower triangular values in Fig. 6d and e is obtained by putting a threshold on $P$-values ($P<0.005$). It can be noticed that cortical cohesion is significantly decreased in the ipsilesional hemisphere but increased in the contralesional hemisphere, shown by the red and blue lines in Fig. 6f. Furthermore, when the E–I balance is globally restored, we observe a significant increase in synchrony in the ipsilesional hemisphere and a decrease in the contralesional hemisphere, shown in red and blue in Fig. 6g. Results for other lesion centers are shown in Supplemental Material, Figure S3.

## Discussion

In this work, we have proposed 2 independent measures SSAs (anatomically self-similar areas) and DSAs (dynamically self-similar areas), using virtual lesion modeling in predicting candidate brain regions that initiate the post-lesion recovery by reestablishing local and global E/I balance. Previous studies (Bullmore and Sporns 2009; Aerts et al. 2016) have explored the critical role of network topology in the context of the brain lesion. However, whether compensatory brain regions could be predicted based on identifying topologically self-similar and dynamical self-similar areas (an equivalence principle) is largely unknown. We have demonstrated the compensatory role of SSAs corresponding to a lesion site/center in restoring E–I balance in the local areas and across the whole brain mediated by the negative feedback form of plasticity. Resetting homeostatic balance results in post-lesion functional re-organization within the surviving cortical areas. The homeostasis mechanisms, governed by the FIC (Deco et al. 2014), are deployed to restore E–I balance within the virtually lesioned brain regions at the group and subject-specific level. We measure the simulation time (called re-adjustment time, $RT$) taken by each region to re-adjust their local inhibitory weights ($J^{\prime}$) during the re-establishment of global homeostatic balance after lesion. Our hypothesis of predicting compensatory brain regions is based on structural and functional equivalence. Further, the observed compensatory utilization of the hemisphere is supported strongly based on SC analysis and FC alteration/rewiring. Our proposed theoretical framework identifies overlapping compensatory brain regions during the early and post-lesion recovery phase across all subjects using 2 independent methods and subject-wise variability. Our results provide the first unified framework behind observing a variety of compensatory brain regions identified by earlier lesion recovery studies (Cao et al. 1999; Winhuisen et al. 2005; Breier et al. 2009; Sharp et al. 2010; Turkeltaub et al. 2012; Szaflarski et al. 2013). The cause and consequence of those identified areas remain a large knowledge gap in the neuroscience literature. We also report that SSAs are more robust and reliable than other network properties widely used in the literature (e.g. clustering coefficient, participation coefficient, node weight) when harnessing region-specific roles in reshaping near-normal functional brain connectivity and dynamics to identify the post-lesion recovery process. The DSAs provide a broader scope for investigating the post-lesion period when SSA is undetermined. The predicted areas (SSAs or DSAs) are independent of the subject’s age and gender. The key compensatory mechanism demonstrated here suggests a CRUH in the emergence of post-lesion coordinated cortical cohesion. Most importantly, the proposed theoretical methods are general and can be applied to broader lesion categories.

### Structural similarity is predictive of functional recovery

Findings from the structural analysis are divided into 2 parts, (i) identification of SSAs and (ii) estimation of DSAs. From the anatomically constrained dynamical mean field model simulation, we have estimated the DSAs, further correlated against the SSAs. Obtaining the correlation between SSAs and DSAs has helped to test our proposed hypothesis. Despite the diversity in inter-individual structural topology, the 2 independent methods (SSAs and DSAs) provide computational machinery for predicting common brain areas with 60–70% overlaps. To check the probability of an estimated region being a potential candidate area for functional recovery, we have measured the probability of appearance (PA) within all 49 subjects in our data. A higher PA corresponding to a predicted area indicates a high chance of being a candidate in all subjects and a high probability of participating in compensation for the damaged brain.

From the derived PA, the SSAs corresponding to the lesion at left POPE (associated with language processing) is identified as CMF, RMF, IFG, PREC and PCNT, and INS in the ipsilesional hemisphere. Contralesional homologous regions are rPOPE and rPTRI. Dynamically similar areas for left POPE are found in both ispi- and contra-lesional hemispheres, including CMF, RMF, PTRI, PREC, and PCNT. Predicted areas belonging to the SSAs and DSAs in both ipsi- and contra-lesional regions suggest a CRUH. The predicted areas are independent of the subject’s age and gender. The identified SSAs from both hemispheres are also reported in earlier studies as essential candidates for complete language recovery after lesion. For example, the recruitment of perilesional tissue (Warburton et al. 1999), as well as contralesional areas (Cao et al. 1999; Winhuisen et al. 2005; Breier et al. 2009; Sharp et al. 2010; Turkeltaub et al. 2012; Szaflarski et al. 2013), and participation of the homologous regions (Weiller et al. 1995; Musso et al. 1999; Rosen et al. 2000) in association with language recovery are well documented largely concurs with our findings. Besides, increased activation of right lesion-homolog IFG has been reported in a subset of patient groups (Rosen et al. 2000).

For a lesion site at the primary motor region, left postcentral gyrus (lPREC), we have predicted candidate areas as CMF, RMF, pars triangularis, superior parietal, superior frontal gyrus, and precuneus in the ipsilesional hemisphere. Moreover, Superior frontal and rostral middle frontal regions from the contralateral hemisphere are found in almost all subjects. Our observations align well with previous findings on ‘motor lesion re-organization in ipsilateral premotor cortex (Connolly et al. 1994; Langhorne et al. 2009), recruitment of contralesional motor areas (Johansen-Berg 2002; Small et al. 2002; Ward et al. 2003). The areas reported by neurological studies found to be crucial in motor function recovery (Nelles et al. 1999). Other motor-related brain regions such as supplementary motor area (SMA), dorsolateral premotor cortex (PMC) and cingulate motor areas (CMA), and insular cortex (Ward 2005) provides necessary compensation to improve motor performance (Calautti and Baron 2003) as documented by previous findings. Also, found to be critical for functional re-organization in the motor recovery (Liu et al. 2011; Griffis et al. 2019) and matches completely with the predicted brain regions based on our proposed computational framework.

In contrast, few regions such as entorhinal (ENT), parahippocampal (PARH), frontal pole (FP), temporal pole (TP), and traverse temporal (TT) regions have displayed significantly less correlation between $JC$ and $RT$ (SSAs and DSAs). The weaker association, in this case, may arise due to their sparse connectivity and weaker anatomical strength. Interestingly, lesions in these brain regions have less impact on overall homeostasis. Thus, the damage to these brain regions may restore the lost E–I balance with comparatively minimal effort. In the above scenario, the adjacent brain regions participated in resetting local and global homeostasis other than SSAs (or DSAs), suggesting local wiring specificity and proximity could be key to initiating the neural compensatory process.

To this end, we mention 2 major aspects of our findings. First, a large overlapping brain region predicted by SSAs and DSAs signifies structurally similar regions primarily participate in the dynamical re-organization process. These areas reset local E–I balance after lesion by modulating their inhibitory weights, thus, displaying the constructive role of SSAs on functional network recovery. It can be concluded that a higher correlation between these 2 independent methods (SSAs and DSAs) arises from the interplay between the structural property and the local inhibitory weights responsible for emergent globally coordinated dynamics. Specifically, an emerging local re-adjustment of inhibitory weights mediates self-organized global brain dynamics during homeostasis. The second aspect of the findings is that the SSAs are identified from the healthy subject’s SC analysis. In contrast, the DSAs are estimated after introducing virtual lesions in the SC matrix. Thus, a methodological advantage is that even if, in the clinical phase, healthy FC is unavailable for a particular patient, the DSAs can be employed as a tool in patient-specific FC to identify candidate compensatory brain areas.

### The compensatory mechanism

In addition, simulation of the virtual lesion model has helped to acquire insight into the dynamic origin of the post-lesion compensatory mechanism. We find that the regions from both ipsi- and contra-lesion, with higher structural similarity to the lesion site, took an extended time to modulate their local inhibitory weights. Besides, the SSAs, from both hemispheres have reduced regional inhibition to balance decreased excitation due to degradation in the excitatory synaptic drive, thus balancing the overall E–I ratio and sustaining the target firing rate of $4$ Hz. As documented earlier, homeostatic plasticity, a mechanism of up-and-down regulation of both the presynaptic release of and the postsynaptic response to neurotransmitters, is essential to maintain a stable set point and near-normal brain condition (Turrigiano and Nelson 2004). Here, the primarily recruited SSAs from the 2 hemispheres suggest the dominant role of structural similarity and rewiring proximity of compensation-related utilization of hemispheres, which have guided homeostatic plasticity-driven compensatory mechanisms in re-organizing post-lesion functional brain network recovery.

### Local E–I balance impacts global FC recovery

Previous studies have reported that changes in excitability affect the local E–I balance of the lesion site and distant cortical networks (Feeney and Baron 1986), known as diaschisis (Carrera and Tononi 2014; Dos et al. 2022), suggesting remote disruptions in FC following lesion impact. These studies have hypothesized mechanisms underlying neuronal remodeling in the perilesional area and contralesional hemisphere after motor cortex infarcts and summarized evidence from previous studies based on analysis of electrophysiological data that demonstrated brain-wide alterations in functional connectivity in both hemispheres, well beyond the infarcted area (Campo et al. 2012; Dos et al. 2022). Our findings based on FC analyses depict reshaping in the coordinated cortical cohesion, which is not limited to the ipsilesional hemisphere but also progresses distant from the damaged area into the contra-lesional hemisphere showing nonlocal effects and completely aligned with the experimental findings from human and animal studies depicting brain regions implicated during the post-lesion functional recovery process.

Our study also observed that an early impact of structural damage results in a specific signature, the reduction in spatiotemporal cortical coherence in the ipsi-lesional hemisphere. On the contrary, the spatiotemporal coherence increases in the contra-lesional hemisphere. In the emergent FC, at E–I balanced state after lesion occurrence, we observed increased synchronous neural activity in the ipsi-lesional site, while a decreased synchronous neural activity in the contra-lesional hemispheres. This seesaw effect in the opposing hemisphere to the lesion center aided functional restoration. The re-organization pattern in the ipsi- and contra-lesional hemispheres is similar in all subjects and for different lesion sites heralding the robustness and consistency of the findings reported here. In response to structural damage, we find that the cortical plasticity mechanism related to E–I homeostasis facilitates FC rewiring in the contra-lesional hemispheres, similar to the previous key observations in Alia et al. (2017). Our proposed computational mechanisms following lesion could be similar to re-organization in pre-infracted to and distant regions from the lesion site that may trigger large-scale remodeling of the cortical networks to compensate for post-lesion deficits (Brown et al. 2009).

In addition, we have reconfirmed our observations using graph-theoretical properties of FC (Gratton et al. 2012; Siegel 2016; Tu et al. 2021) such as transitivity, path length, modularity, and global efficiency, which are interpreted in terms of lesion impacts, and FC recovery. Due to lesions, the biological perturbation reshapes the healthy normative pattern in FC. The loss of inter-area excitatory synapses predominantly affects coordinated neural dynamics, which can be observed from the segregated functional network captured by the increased modularity and decreased global efficiency. However, the damaged brain tries to adapt to the global changes in functions caused by the homeostatic imbalance across the whole brain. Our $\textit{in-silico}$ investigation suggests that the adaptive mechanism compensates for the lost E–I homeostasis, primarily driven by the modulation of inhibition at the local level, mainly within the SSAs (or DSAs) identified in this work.

Gradual resetting of neural activity to regain the near-normal function is confirmed by the decreased modularity and increased global efficiency concerning the altered FCs. The FC integration has compensated segregation of FC after the damage to underlying structural connections. This is in concurrence with the previous findings of 2 model-based measures: ”integration,” a theory-based on graph theoretical measure obtained from functional connectivity, which measures the connectedness of brain networks, and ”information capacity,” an information-theoretic measure, representative of the segregative ability of the brain networks to encode distinct stimuli (Adhikari et al. 2017). This further resets healthy dynamical repertoire driven by the negative feedback-mediated form of plasticity leading to re-organization on biological timescale. Further, it is well explained in earlier studies (Bütefisch et al. 2003; Carmichael 2003) that the global homeostasis is balanced by increasing excitability in the areas near and distant to the lesion center, suggesting a direct correlation between the E–I balance and global cortical dynamics (Roy et al. 2014), which can be one crucial aspect in proper lesion recovery.

### Limitations

There are also limitations of this study. (i) A precise mapping between the accurate lesion biological time scale and simulation time is still being determined. Therefore, we cannot predict the time scale of the actual recovery process. Mapping real-time-scale with simulation time can bring us closer to uncovering the true recovery mechanism and will have excellent translational value. We are currently investigating this mapping in another research work and out of the scope of this study. (ii) The FIC mediates the homeostasis mechanism. However, other feedback-mediated mechanisms may be incorporated into the model to verify increased excitability due to lesions in individual subjects, which this work does not sufficiently explore. (iii) We did not incorporate the effect of lesion volume in this study. The amount of lesion volume and spread are essential factors in FC re-organization and recovery, which are not addressed here. (iv) Directed FC also holds the key to understanding how information flow alters following lesion and during recovery, which future studies may explore, (v) Finally, how FC is reshaped based on longitudinal data can validate present findings in a more nuanced fashion and establish a stronger link between lesion recovery and functional re-organization elucidating region-specific roles. One of the potential measures to assess the changes could be the use of dynamic functional connectivity (Sastry et al. 2023). However, FCD largely captures temporal correlations at the expense of spatial/ROI-specific information. Hence, of little value in predicting SSAs in our study context based on virtual lesions. Furthermore, the mathematical formalism becomes unnecessarily complex to relate dJJ (change in local inhibitory weights) post-lesion recovery mechanisms to track DSAs. This renders FCD less useful as a measure in the context of our primary objective of predicting compensatory brain areas in large-scale brain networks. (vi) The parcellation used in this study is Desikan–Killiany (DK) parcellation as in by Schirner et al. (2015), which consists of 68 cortical ROIs with 34 ROIs in each hemisphere. SC matrices generated from each subject’s MRI data are averaged elementwise to obtain an SC matrix. The connectivity strength between each pair of 68 areas represents how one area can influence other areas in the context of a specific model (refer to Computational model simulating whole brain resting dynamics section). While using the DK atlas helps simplify numerical simulations, a higher-resolution parcellation would likely improve predictions for SSAs and DSAs at a much finer spatial scale.

### Conclusion and future aspects

In conclusion, we envision a novel method to identify potential candidate areas responsible for resetting E–I homeostasis as possible compensatory mechanisms resulting in near-normal functional brain network recovery. A fundamental open question in the literature is how the non-lesioned brain adapts to the post-injury functional recovery process and whether those areas could be predicted using a systematic theoretical framework. Although the lesion recovery process may be complex, the current study provides a general framework elucidating that brain recovery involves the utilization of an equivalence principle based on structural and dynamic similarity to tackle a wide variety of lesions. Future studies could use controllability theory to narrow the DSA (SSA) estimation into a specific region on the directed FC network. Those studies could further pinpoint whether a DSA (SSA) driven information flow pattern exists in the surviving cortex. Furthermore, how do the candidate brain areas help information fidelity during the brain network recovery?

## Author contributions

Priyanka Chakraborty (Conceptualization, Data curation, Formal analysis, Investigation, Methodology, Resources, Validation, Visualization, Writing—original draft, Writing—review & editing). Suman Saha (Conceptualization, Formal analysis, Investigation, Methodology, Validation, Visualization, Writing—original draft, Writing—review & editing). Gustavo Deco (Writing—original draft, Writing—review & editing). Arpan Banerjee (Formal analysis, Funding acquisition, Investigation, Methodology, Project administration, Resources, Supervision, Validation, Visualization, Writing—original draft, Writing—review & editing). Dipanjan Roy (Conceptualization, Data curation, Formal analysis, Funding acquisition, Investigation, Methodology, Project administration, Resources, Supervision, Validation, Visualization, Writing—original draft, Writing—review & editing).

## Supplementary Material

ls_supplyement_542023_tgad012

ls_PC_SS_GD_AB_DR_final_accepted_tgad012

## Data Availability

Our analysis code is available on GitHub (https://github.com/dynamicdip/Virtual-Lesion-Project-CBDL).

## Supplemental material

Supplemental Material contains Tables S1–S3, Figures S1–S7, details of the empirical data, DMF model description and SI references. Table S1 shows model parameter descriptions and default values. Table S2 contains region names and abbreviations. Table S3 contains list of SSAs and DSAs for different lesion centers. Predicted SSAs and DSAs for 10 subjects in Figure S1. Association between structural and dynamical measures for different lesion centers over all subjects in Figure S2. Details of the regions with lower connectivity in Figure S3. Figure S4 shows results for group-level analysis on SSAs and DSAs. ROI-wise FC analysis over all subjects for different lesion centers is shown in Figure S5. Correlation between JC and other network properties of SC in Figure S6. Figure S7 depicts FC properties under 3 given conditions.

## CRediT author statement

The research was designed by PC, SS, and DR. Methodology was implemented by PC, SS, AB, and DR. Investigation was carried out by PC and SS. Supervision and funding for the project was provided by AB and DR. Original draft was written by PC, SS, and DR. Subsequent drafts were reviewed and edited by PC, SS, AB, GD, and DR. PC and SS contributed equally to this work.

## References

[ref1] Adhikari MH, Beharelle AR, Griffa A, Hagmann P, Solodkin A, McIntosh AR, Small SL, Deco G. Computational modeling of resting-state activity demonstrates markers of normalcy in children with prenatal or perinatal stroke. J Neurosci. 2015: 35(23): 8914–8924. 26063923 10.1523/JNEUROSCI.4560-14.2015 PMC4589568

[ref2] Adhikari MH, Hacker CD, Siegel JS, Griffa A, Hagmann P, Deco G, Corbetta M. Decreased integration and information capacity in stroke measured by whole brain models of resting state activity. Brain. 2017: 140(4): 1068–1085. 28334882 10.1093/brain/awx021 PMC6075429

[ref3] Aerts H, Fias W, Caeyenberghs K, Marinazzo D. Brain networks under attack: robustness properties and the impact of lesions. Brain. 2016: 139(12): 3063–3083. 27497487 10.1093/brain/aww194

[ref4] Alia C, Cangi D, Massa V, Salluzzo M, Vignozzi L, Caleo M, Spalletti C. Neuroplastic changes following brain ischemia and their contribution to stroke recovery: novel approaches in neurorehabilitation. Front Cell Neurosci. 2017: 11:76. 28360842 10.3389/fncel.2017.00076 PMC5352696

[ref5] Alstott J, Breakspear M, Hagmann P, Cammoun L, Sporns O. Modeling the impact of lesions in the human brain. PLoS Comput Biol. 2009: 5(6):e1000408. 10.1371/journal.pcbi.1000408 PMC2688028 19521503

[ref6] Arundine M, Tymianski M. Molecular mechanisms of glutamate-dependent neurodegeneration in ischemia and traumatic brain injury. Cell Molec Life Sci CMLS. 2004: 61(6): 657–668. 15052409 10.1007/s00018-003-3319-x PMC11138528

[ref7] Arundine M . Enhanced vulnerability to nmda toxicity in sublethal traumatic neuronal injury in vitro. J Neurotrauma. 2003: 20(12): 1377–1395. 14748985 10.1089/089771503322686166

[ref8] Besancon E . Beyond nmda and ampa glutamate receptors: emerging mechanisms for ionic imbalance and cell death in stroke. Trends Pharma Sci. 2008: 29(5): 268–275. 10.1016/j.tips.2008.02.003 18384889

[ref9] Bolay H, Gürsoy-Özdemir Y, Sara Y, Onur R, Can A, Dalkara T. Persistent defect in transmitter release and synapsin phosphorylation in cerebral cortex after transient moderate ischemic injury. Stroke. 2002: 33(5): 1369–1375. 11988617 10.1161/01.str.0000013708.54623.de

[ref10] Breier JI, Juranek J, Maher LM, Schmadeke S, Men D, Papanicolaou AC. Behavioral and neurophysiologic response to therapy for chronic aphasia. Arch Phys Med Rehabil. 2009: 90(12): 2026–2033. 19969164 10.1016/j.apmr.2009.08.144 PMC3068866

[ref11] Brown CE, Aminoltejari K, Erb H, Winship IR, Murphy TH. In vivo voltage-sensitive dye imaging in adult mice reveals that somatosensory maps lost to stroke are replaced over weeks by new structural and functional circuits with prolonged modes of activation within both the peri-infarct zone and distant sites. J Neurosci. 2009: 29(6): 1719–1734. 19211879 10.1523/JNEUROSCI.4249-08.2009 PMC6666293

[ref12] Buchkremer-Ratzmann I, August M, Hagemann G, Witte OW. Electrophysiological transcortical diaschisis after cortical photothrombosis in rat brain. Stroke. 1996: 27(6): 1105–1111. 8650722 10.1161/01.str.27.6.1105

[ref13] Bullmore E, Sporns O. Complex brain networks: graph theoretical analysis of structural and functional systems. Nat Rev Neurosci. 2009: 10(3): 186–198. 19190637 10.1038/nrn2575

[ref14] Burns BD, Webb A. The spontaneous activity of neurones in the cat’s cerebral cortex. Proc of the Roy Soc Lond Ser B Bio Sci. 1976: 194(1115): 211–223. 10.1098/rspb.1976.0074 11486

[ref15] Bütefisch CM, Netz J, Wessling M, Seitz RJ, Hömberg V. Remote changes in cortical excitability after stroke. Brain. 2003: 126(2): 470–481. 12538413 10.1093/brain/awg044

[ref16] Cabral J, Hugues E, Kringelbach ML, Deco G. Modeling the outcome of structural disconnection on resting-state functional connectivity. NeuroImage. 2012: 62(3): 1342–1353. 22705375 10.1016/j.neuroimage.2012.06.007

[ref17] Caeyenberghs K, Leemans A, Leunissen I, Michiels K, Swinnen SP. Topological correlations of structural and functional networks in patients with traumatic brain injury. Front Hum Neurosci. 2013: 7:726. 24204337 10.3389/fnhum.2013.00726 PMC3817367

[ref18] Calautti C, Baron J-C. Functional neuroimaging studies of motor recovery after stroke in adults: a review. Stroke. 2003: 34(6): 1553–1566. 12738893 10.1161/01.STR.0000071761.36075.A6

[ref19] Campo P, Garrido MI, Moran RJ, Maestu F, Garcia-Morales I, Gil-Nagel A, del Pozo F, Dolan RJ, Friston KJ. Remote effects of hippocampal sclerosis on effective connectivity during working memory encoding: a case of connectional diaschisis? Cereb Cortex. 2012: 22(6): 1225–1236. 21810779 10.1093/cercor/bhr201 PMC3357177

[ref20] Cao Y, Vikingstad EM, George KP, Johnson AF, Welch K. Cortical language activation in stroke patients recovering from aphasia with functional mri. Stroke. 1999: 30(11): 2331–2340. 10548667 10.1161/01.str.30.11.2331

[ref21] Carmichael ST . Plasticity of cortical projections after stroke. Neurosct. 2003: 9(1): 64–75. 10.1177/1073858402239592 12580341

[ref22] Carmichael ST, Chesselet M-F. Synchronous neuronal activity is a signal for axonal sprouting after cortical lesions in the adult. J Neurosci. 2002: 22(14): 6062–6070. 12122067 10.1523/JNEUROSCI.22-14-06062.2002 PMC6757933

[ref23] Carmichael ST, Tatsukawa K, Katsman D, Tsuyuguchi N, Kornblum HI. Evolution of diaschisis in a focal stroke model. Stroke. 2004: 35(3): 758–763. 14963280 10.1161/01.STR.0000117235.11156.55

[ref24] Carrera E, Tononi G. Diaschisis: past, present, future. Brain. 2014: 137(9): 2408–2422. 24871646 10.1093/brain/awu101

[ref25] Cirstea M, Levin MF. Compensatory strategies for reaching in stroke. Brain. 2000: 123(5): 940–953. 10775539 10.1093/brain/123.5.940

[ref26] Connolly AM, Dodson WE, Prensky AL, Rust RS. Course and outcome of acute cerebellar ataxia. Annal Neurol. 1994: 35(6): 673–679. 8210223 10.1002/ana.410350607

[ref27] Crofts JJ, Higham DJ, Bosnell R, Jbabdi S, Matthews PM, Behrens T, Johansen-Berg H. Network analysis detects changes in the contralesional hemisphere following stroke. NeuroImage. 2011: 54(1): 161–169. 20728543 10.1016/j.neuroimage.2010.08.032 PMC3677803

[ref81] Dall’Acqua P, Johannes S, Mica L, Simmen H-P, Glaab R, Fandino J, Schwendinger M, Meier C, Ulbrich EJ, Müller A. Functional and structural network recovery after mild traumatic brain injury: a 1-year longitudinal study. Front Hum Neurosci. 2017: 11:280. 28611614 10.3389/fnhum.2017.00280 PMC5447750

[ref28] Deco G, Jirsa VK. Ongoing cortical activity at rest: criticality, multistability, and ghost attractors. J Neurosci. 2012: 32(10): 3366–3375. 22399758 10.1523/JNEUROSCI.2523-11.2012 PMC6621046

[ref29] Deco G, Jirsa VK, Robinson PA, Breakspear M, Friston K. The dynamic brain: from spiking neurons to neural masses and cortical fields. PLoS Comput Biol. 2008: 4(8):e1000092. 10.1371/journal.pcbi.1000092 PMC2519166 18769680

[ref30] Deco G, Ponce-Alvarez A, Hagmann P, Romani GL, Mantini D, Corbetta M. How local excitation–inhibition ratio impacts the whole brain dynamics. J Neurosci. 2014: 34(23): 7886–7898. 24899711 10.1523/JNEUROSCI.5068-13.2014 PMC4044249

[ref31] Desikan RS, Ségonne F, Fischl B, et al. An automated labeling system for subdividing the human cerebral cortex on mri scans into gyral based regions of interest. NeuroImage. 2006: 31(3): 968–980. 16530430 10.1016/j.neuroimage.2006.01.021

[ref32] Dos, Santos FP, Verschure PF. Excitatory-inhibitory homeostasis and diaschisis: tying the local and global scales in the post-stroke cortex. Front Syst Neurosci. 2022: 15:806544. 10.3389/fnsys.2021.806544 PMC8785563 35082606

[ref33] Duffau H, Capelle L, Denvil D, Sichez N, Gatignol P, Lopes M, Mitchell M, Sichez J, Van Effenterre R. Functional recovery after surgical resection of low grade gliomas in eloquent brain: hypothesis of brain compensation. J Neurol Neurosurg Psychiatry. 2003: 74(7): 901–907. 12810776 10.1136/jnnp.74.7.901 PMC1738559

[ref34] Feeney DM, Baron J-C. Diaschisis Stroke. 1986: 17(5): 817–830. 3532434 10.1161/01.str.17.5.817

[ref35] Gao T, Pulsinelli W, Xu Z. Changes in membrane properties of ca1 pyramidal neurons after transient forebrain ischemia in vivo. Neuroscience. 1999: 90(3): 771–780. 10218778 10.1016/s0306-4522(98)00493-x

[ref36] Gerloff C, Bushara K, Sailer A, et al. Multimodal imaging of brain reorganization in motor areas of the contralesional hemisphere of well recovered patients after capsular stroke. Brain. 2006: 129(3): 791–808. 16364955 10.1093/brain/awh713

[ref37] Gratton C, Nomura EM, Pérez F, D’Esposito M. Focal brain lesions to critical locations cause widespread disruption of the modular organization of the brain. J Cogn Neurosci. 2012: 24(6): 1275–1285. 22401285 10.1162/jocn_a_00222 PMC3575518

[ref38] Griffis JC, Metcalf NV, Corbetta M, Shulman GL. Structural disconnections explain brain network dysfunction after stroke. Cell Rep. 2019: 28(10): 2527–2540. 31484066 10.1016/j.celrep.2019.07.100 PMC7032047

[ref1h] Haider B, Häusser M, Carandini M. Inhibition dominates sensory responses in the awake cortex. Nature. Nature Publishing Group UK London, 2013: 493(7430): 97–100. 10.1038/nature11665 PMC3537822 23172139

[ref39] Hilgetag C-C, Burns GAPC, O’Neill MA, Scannell JW, Young MP. Anatomical connectivity defines the organization of clusters of cortical areas in the macaque and the cat. Phil Trans Roy Soc Lond Ser B: Bio Sci. 2000: 355(1393): 91–110. 10703046 10.1098/rstb.2000.0551 PMC1692723

[ref40] Hossmann K-A . Pathophysiology and therapy of experimental stroke. Cell Mole Neurobio. 2006: 26(7): 1055–1081. 10.1007/s10571-006-9008-1 PMC11520628 16710759

[ref41] Huynh W, Vucic S, Krishnan AV, Lin CS, Kiernan MC. Exploring the evolution of cortical excitability following acute stroke. Neurorehabil Neural Repair. 2016: 30(3): 244–257. 26150146 10.1177/1545968315593804

[ref42] Johansen-Berg H . The role of ipsilateral premotor cortex in hand movement after stroke. Proc Natl Acad Sci. 2002: 99(22): 14518–14523. 12376621 10.1073/pnas.222536799 PMC137915

[ref43] Kolb B, Whishaw IQ. Brain plasticity and behavior. Annual Rev Psych. 1998: 49(1): 43–64. 10.1146/annurev.psych.49.1.43 9496621

[ref44] Langhorne P, Coupar F, Pollock A. Motor recovery after stroke: a systematic review. Lancet Neurol. 2009: 8(8): 741–754. 19608100 10.1016/S1474-4422(09)70150-4

[ref45] Lashley KS. In search of the engram. In Society for Experimental Biology, Physiological mechanisms in animal behavior. (Society’s Symposium IV.). Academic Press, (pp. 454–482). 1950.

[ref46] Liu Y-Y, Slotine J-J, Barabási A-L. Controllability of complex networks. Nature. 2011: 473(7346): 167–173. 21562557 10.1038/nature10011

[ref47] Maeda M, Miyazaki M. Control of ICP and the cerebrovascular bed by the cholinergic basal forebrain. In: Marmarou, Bullock A, Avezaat R, et al. (eds). Intracranial pressure and neuromonitoring in brain injury. Vienna: Springer; 1998. 71, p. 293–296 10.1007/978-3-7091-6475-4_85 9779211

[ref48] Mitchell JF, Sundberg KA, Reynolds JH. Differential attention-dependent response modulation across cell classes in macaque visual area v4. Neuron. 2007: 55(1): 131–141. 17610822 10.1016/j.neuron.2007.06.018

[ref49] Moreira da Silva N, Cowie CJ, Blamire AM, Forsyth R, Taylor PN. Investigating brain network changes and their association with cognitive recovery after traumatic brain injury: a longitudinal analysis. Front Neurol. 2020: 11:369. 32581989 10.3389/fneur.2020.00369 PMC7296134

[ref50] Murphy TH, Corbett D. Plasticity during stroke recovery: from synapse to behaviour. Nat Rev Neurosci. 2009: 10(12): 861–872. 19888284 10.1038/nrn2735

[ref51] Murphy TH, Li P, Betts K, Liu R. Two-photon imaging of stroke onset in vivo reveals that nmda-receptor independent ischemic depolarization is the major cause of rapid reversible damage to dendrites and spines. J Neurosci. 2008: 28(7): 1756–1772. 18272696 10.1523/JNEUROSCI.5128-07.2008 PMC6671530

[ref52] Musso M, Weiller C, Kiebel S, Müller SP, Bülau P, Rijntjes M. Training-induced brain plasticity in aphasia. Brain. 1999: 122(9): 1781–1790. 10468516 10.1093/brain/122.9.1781

[ref53] Musuka TD, Wilton SB, Traboulsi M, Hill MD. Diagnosis and management of acute ischemic stroke: speed is critical. CMAJ. 2015: 187(12): 887–893. 26243819 10.1503/cmaj.140355 PMC4562827

[ref54] Nelles G, Spiekermann G, Jueptner M, Leonhardt G, Müller S, Gerhard H, Diener HC. Evolution of functional reorganization in hemiplegic stroke: a serial positron emission tomographic activation study. Annal Neurol. 1999: 46(6): 901–909. 10589543 10.1002/1531-8249(199912)46:6<901::aid-ana13>3.0.co;2-7

[ref55] Nudo RJ . Postinfarct cortical plasticity and behavioral recovery. Stroke. 2007: 38(2): 840–845. 17261749 10.1161/01.STR.0000247943.12887.d2

[ref56] O’Reilly JX, Croxson PL, Jbabdi S, et al. Causal effect of disconnection lesions on interhemispheric functional connectivity in rhesus monkeys. Proc Natl Acad Sci. 2013: 110(34): 13982–13987. 23924609 10.1073/pnas.1305062110 PMC3752223

[ref57] Rosen H, Petersen S, Linenweber M, Snyder A, White D, Chapman L, Dromerick A, Fiez J, Corbetta M. Neural correlates of recovery from aphasia after damage to left inferior frontal cortex. Neurology. 2000: 55(12): 1883–1894. 11134389 10.1212/wnl.55.12.1883

[ref58] Roy D, Sigala R, Breakspear M, McIntosh AR, Jirsa VK, Deco G, Ritter P. Using the virtual brain to reveal the role of oscillations and plasticity in shaping brain’s dynamical landscape. Brain Connectivity. 2014: 4(10): 791–811. 25131838 10.1089/brain.2014.0252

[ref59] Sastry NC, Roy D, Banerjee A. Stability of sensorimotor network sculpts the dynamic repertoire of resting state over lifespan. Cereb Cortex. 2023: 33(4): 1246–1262. 35368068 10.1093/cercor/bhac133 PMC9930636

[ref60] Schirner M, Rothmeier S, Jirsa VK, McIntosh AR, Ritter P. An automated pipeline for constructing personalized virtual brains from multimodal neuroimaging data. NeuroImage. 2015: 117:343–357. 25837600 10.1016/j.neuroimage.2015.03.055

[ref61] Shadlen MN, Newsome WT. The variable discharge of cortical neurons: implications for connectivity, computation, and information coding. J Neurosci. 1998: 18(10): 3870–3896. 9570816 10.1523/JNEUROSCI.18-10-03870.1998 PMC6793166

[ref62] Sharp DJ, Turkheimer FE, Bose SK, Scott SK, Wise RJ. Increased frontoparietal integration after stroke and cognitive recovery. Annal Neurol. 2010: 68(5): 753–756. 20687116 10.1002/ana.21866

[ref63] Siegel JS . Disruptions of network connectivity predict impairment in multiple behavioral domains after stroke. Proc Natl Acad Sci. 2016: 113(30): E4367–E4376. 27402738 10.1073/pnas.1521083113 PMC4968743

[ref64] Small SL, Hlustik P, Noll DC, Genovese C, Solodkin A. Cerebellar hemispheric activation ipsilateral to the paretic hand correlates with functional recovery after stroke. Brain. 2002: 125(7): 1544–1557. 12077004 10.1093/brain/awf148

[ref65] Szaflarski JP, Allendorfer JB, Banks C, Vannest J, Holland SK. Recovered vs. not-recovered from post-stroke aphasia: the contributions from the dominant and non-dominant hemispheres. Rest Neuro Neurosci. 2013: 31(4): 347–360. 10.3233/RNN-120267 PMC3701454 23482065

[ref66] Tao Y, Rapp B. Investigating the network consequences of focal brain lesions through comparisons of real and simulated lesions. Sci Rep. 2021: 11(1): 1–17. 33500494 10.1038/s41598-021-81107-9 PMC7838400

[ref67] Tu W, Ma Z, Zhang N. Brain network reorganization after targeted attack at a hub region. NeuroImage. 2021: 237:118219. 10.1016/j.neuroimage.2021.118219 PMC8289586 34052466

[ref68] Turkeltaub PE, Coslett HB, Thomas AL, Faseyitan O, Benson J, Norise C, Hamilton RH. The right hemisphere is not unitary in its role in aphasia recovery. Cortex. 2012: 48(9): 1179–1186. 21794852 10.1016/j.cortex.2011.06.010 PMC3221765

[ref69] Turrigiano GG, Nelson SB. Homeostatic plasticity in the developing nervous system. Nat Rev Neurosci. 2004: 5(2): 97–107. 14735113 10.1038/nrn1327

[ref70] Vattikonda A, Surampudi BR, Banerjee A, Deco G, Roy D. Does the regulation of local excitation–inhibition balance aid in recovery of functional connectivity? A computational account. NeuroImage. 2016: 136:57–67. 27177761 10.1016/j.neuroimage.2016.05.002

[ref71] Wang J-H . Short-term cerebral ischemia causes the dysfunction of interneurons and more excitation of pyramidal neurons in rats. Brain Res Bull. 2003: 60(1–2): 53–58. 12725892 10.1016/s0361-9230(03)00026-1

[ref72] Warburton E, Price CJ, Swinburn K, Wise RJS. Mechanisms of recovery from aphasia: evidence from positron emission tomography studies. J Neurol Psychiatry. 1999: 66(2): 155–161. 10.1136/jnnp.66.2.155 PMC1736204 10071093

[ref73] Ward N . Mechanisms underlying recovery of motor function after stroke. Postgrad Med J. 2005: 81(958): 510–514. 16085742 10.1136/pgmj.2004.030809 PMC1743338

[ref74] Ward N, Brown M, Thompson A, Frackowiak R. Neural correlates of motor recovery after stroke: a longitudinal fmri study. Brain. 2003: 126(11): 2476–2496. 12937084 10.1093/brain/awg245 PMC3717457

[ref75] Willer C, Ramsay SC, Wise RJS, Friston KJ, Frackwiak RSJ. Individual patterns of functional reorganization in the human cerebral cortex after capsular infarction. Annal Neurol. 1993: 33(2): 181–189. 8434880 10.1002/ana.410330208

[ref76] Weiller C, Isensee C, Rijntjes M, Huber W, Müller S, Bier D, Dutschka K, Woods RP, Noth J, Diener HC. Recovery from wernicke’s aphasia: a positron emission tomographic study. Annal Neurol. 1995: 37(6): 723–732. 7778845 10.1002/ana.410370605

[ref77] Werner C, Engelhard K. Pathophysiology of traumatic brain injury. British Journal of Anaesthesia. 2007: 99(1): 4–9. 10.1093/bja/aem131. 17573392

[ref78] Winhuisen L, Thiel A, Schumacher B, Kessler J, Rudolf J, Haupt WF, Heiss WD. Role of the contralateral inferior frontal gyrus in recovery of language function in poststroke aphasia: a combined repetitive transcranial magnetic stimulation and positron emission tomography study. Stroke. 2005: 36(8): 1759–1763. 16020770 10.1161/01.STR.0000174487.81126.ef

[ref79] Witte OW . Lesion-induced plasticity as a potential mechanism for recovery and rehabilitative training. Curr Opin Neuro. 1998: 11(6): 655–662. 10.1097/00019052-199812000-00008 9870133

[ref80] Wong K-F, Wang X-J. A recurrent network mechanism of time integration in perceptual decisions. J Neurosci. 2006: 26(4): 1314–1328. 16436619 10.1523/JNEUROSCI.3733-05.2006 PMC6674568

