# Supplementary material for: Structural-and-dynamical similarity predicts compensatory brain areas driving the post-lesion functional recovery mechanism
Source: Cereb Cortex Commun. 2023 Jul 17;4(3):tgad012. doi: 10.1093/texcom/tgad012 (PMC10409568; doi:10.1093/texcom/tgad012)
Supplement: ls_supplyement_542023_tgad012 [file ls_supplyement_542023_tgad012.zip › ls_supplyement_542023_tgad012.pdf]

Structural-and-functional equivalence principle predicts compensatory brain areas driving the post-lesion functional recovery mechanism

Priyanka Chakraborty*[1], Suman Saha*[1], Gustavo Deco[2,3,4,5], Arpan Banerjee[1], Dipanjan Roy†[6]
† *Dipanjan Roy, E-mail: droy@iitj.ac.in*

Empirical data

Participants

MRI and resting-state functional MRI data from 49 healthy subjects (31 females), ages ranging from 18 to 80 years (mean age: 41.55 years; standard deviation: 18.44 years), have been collected at Berlin Center for Advanced Imaging, Charité University Medicine, Berlin, Germany [Schirner and et al., 2015]. The participants are healthy, and no history of neurologic or psychiatric conditions was reported in [Schirner and et al., 2015]. All participants gave written informed consent to the group [Schirner and et al., 2015], and the study was performed under the compliance of laws and guidelines approved by the ethics committee of Charité University, Berlin, Germany.

Anatomical connectivity

Resting state MRI, diffusion-weighted MRI, and functional MRI are performed using a 3 Tesla Siemens Tim Trio MR scanner and a 12-channel Siemens head coil. Detailed information on data acquisition parameters is found in [Schirner and et al., 2015]. We did not process the raw data. The data was pre-processed, and structural connectome was generated previously, using the pipeline by Schirner et al. [Schirner and et al., 2015]. Cortical grey matter parcellation of 34 regions of interest(ROI) in each hemisphere is considered following Desikan-Killiany parcellation [Desikan et al., 2006]. The SI Appendix, Table S1, shows all the regions of interest (ROIs) with abbreviations.

Empirical functional connectivity

Participants are subjected to a functional MRI scan in eyes-closed awake resting-state condition. The resting-state BOLD activity is recorded for 22 minutes (TR=2 sec). Pre-processing steps are given in SI Appendix. After pre-processing, aggregated BOLD time series of each region is z-transformed. The pairwise Pearson correlation coefficient is computed for each subject's resting-state functional connectivity (rs-FC) matrix.

Preprocessing of empirical data

Structural connectivity

Each subject's empirical structural connectivity (SC) was generated using the pipeline described by Schiner et al. [Schirner and et al., 2015]. Main pre-processing steps for T1 anatomical images involved skull stripping, removal of non-brain tissue, brain mask generation, cortical reconstruction, motion correction, intensity normalization, WM, subcortical segmentation, cortical tessellation generating GM-WM and GM-pia interface surface-triangulations and probabilistic atlas based cortical and subcortical parcellation. Cortical grey matter parcellation of 34 regions of interest(ROI) in each hemisphere was undertaken following Desikan-Killiany parcellation [Desikan et al., 2006]. The probabilistic tractography algorithm estimated the connection strength (a value ranging from 0 to 1) between each pair of ROIs. SC matrices were generated from each subjecTs MRI data and then summed element-wise to obtain an averaged SC matrix. The

connection of a region to itself was set to 0 in the SC matrix for the simulations. motion correction and eddy current correction (ECC), the b0 image is linearly registered to the subject's anatomical T1-weighted image, and the resulting registration rule is used to transform the high-resolution mask volumes from the anatomical space to the subject's diffusion space. MRTrix has been used to extract gradient vectors and values (b-table). DW-MRI data were pre-processed using FREESURFER. The pre-processing steps for the diffusion MRI data were eddy current and motion correction with re-orientation of b-vectors (b-zero image was linearly registered to the subject's anatomical T1-weighted image). Then, fiber-response function estimation has been done. The fiber orientation distribution function (fODF) for each image voxel has been computed based on constrained spherical deconvolution (CSD) in MRTrix. Structural connectome is the count of tracks between any given pair of ROIs. SC is normalized and symmetric.

Functional connectivity

Each subject's empirical functional connectivity (FC) was computed using the pipeline described by Schiner et al. [Schirner and et al., 2015]. To generate the functional connectivity (FC) matrices, pre-processing steps are as follows: 1) raw fMRI DICOM files were converted into a single 4D Nifti image file. 2) FSL's FEAT pipeline is used to perform the following operations: a) deleting the first five images of the series to exclude possible saturation effects in the images, b) high-pass temporal filtering (100 seconds high-pass filter), c) motion correction, d) brain extraction and e) a 6 DOF linear registration to the MNI space. 3) BOLD signals are registered to the subject's T1-weighted images and parcellated according to FREESURFER's cortical segmentation (Desikan-Killiany (DK) atlas [Desikan et al., 2006]). 4) The inverted mapping rule mapped Anatomical segmentation onto the functional space. 5) Average BOLD signal time series for each ROI were generated by computing the mean of all voxel time series of each region. 6) From the region-wise aggregated BOLD data, FC matrices were computed within MATLAB using pairwise mutual information (on z-transformed data), and Pearson's linear correlation coefficient as FC metrics.

Dynamic mean field (DMF) model

We use a reduced dynamic mean field (DMF) model [Wong and Wang, 2006] to engender lesion effects. The DMF approximates a spiking network model [Deco and Jirsa, 2012, Deco et al., 2014] consisting of populations of excitatory and inhibitory neurons with excitatory NMDA synapses and inhibitory GABA synapses. DMF is described by a set of coupled nonlinear stochastic differential equations given below,

$$
\begin{aligned}
I_i^{(E)} &= w_E I_0 + w_+ J_N S_i^{(E)} + G J_N \sum_{j=1}^{N} C_{ij} S_j^{(E)} - J_i S_i^{(I)} \\
I_i^{(I)} &= w_I I_0 + J_N S_i^{(E)} - w_{II} S_i^{(I)} \\
r_i^{(E)} &= \frac{a_E I_i^{(E)} - b_E}{1 - e^{-d_E\left(a_E I_i^{(E)} - b_E\right)}} \\
r_i^{(I)} &= \frac{a_I I_i^{(I)} - b_I}{1 - e^{-d_I\left(a_I I_i^{(I)} - b_I\right)}} \\
\frac{dS_i^{(E)}}{dt} &= -\frac{S_i^{(E)}}{\tau_E} + \left(1 - S_i^{(E)}\right) \gamma r_i^{(E)} + \sigma \nu_i(t) \\
\frac{dS_i^{(I)}}{dt} &= -\frac{S_i^{(I)}}{\tau_I} + r_i^{(I)} + \sigma \nu_i(t),
\end{aligned} \tag{1}
$$

where $I_i^{E,I}$ is the input current to area i and superscripts represent excitatory (E) and inhibitory(I) populations in that area. $r_i^{E,I}$ is the population firing rate of excitatory or inhibitory populations of area i. $S_i^{E,I}$ is the average excitatory (inhibitory) synaptic gating variable of area i. I_0 is the effective external input scaled by w_E and w_I for excitatory and inhibitory populations. w_+ is the local excitatory recurrence, J_N is the excitatory synaptic coupling, and J_i is the local feedback inhibitory synaptic coupling. w_{II} is the local inhibitory recurrence. C_{ij} is the i, j^{th} entry in the SC matrix, obtained from diffusion imaging, that scales the long-range excitatory currents between j^{th} and i^{th} regions. G represents global coupling strength which scales long-range excitatory connections. Descriptions of the parameters and their default values are given in Table S2. To find optimal G, the DMF model is simulated for different values of G. The optimal value of G is chosen based on the highest correlation between empirical and simulated FC, when the excitatory firing rate sustains at ∼4Hz within all brain regions [Burns and Webb, 1976, Shadlen and Newsome, 1998]. Stochasticity is incorporated into the two gating variables by additive white Gaussian noise, $\sigma\nu_i(t)$, where σ is the noise intensity.

Table 1: Model parameters and their values

Parameter	Description	Value/ Units
I_0	External input current	0.382 nA
w_E	Weight for excitatory populations	1
J_N	Long-range excitatory synaptic coupling constant	0.15nA
w_+	Strength of local excitatory recurrent connections	1.4
a_E	Parameter for input-output function	310nC^{-1}
b_E	Parameter for input-output function	125 Hz
d_E	Parameter for input-output function	0.16s
w_I	Weight for inhibitory populations	0.7
a_I	Parameter for input-output function	$615\ \text{nC}^{-1}$
b_I	Parameter for input-output function	177 Hz
d_I	Parameter for input-output function	0.087s
w_{II}	Strength of local inhibitory recurrent connections	1nA
γ	Learning rate	0.641
τ_E	Excitatory time constant	100 ms
τ_I	Inhibitory time constant	10 ms
σ	Noise amplitude	0.001nA
G	Global coupling strength	0.55

Definitions and descriptions

Defining and describing the terminologies related to the study is worthwhile before drawing the pipeline and workflow. The definitions of the Jaccard coefficient, virtual lesion, DMF model, virtual lesion model, re-adjusted inhibitory weights, and time to reach E-I balance are presented below:

Feedback inhibition control (FIC)

FIC algorithm, proposed by Deco et al. [Deco et al., 2014], is a recursive process to establish and maintain E-I balance in individual and across all cortical subunits. By the term E-I balance, it means that the average input current (I_i^E) to an excitatory pool of ith region is equal to $\frac{b_E}{a_E} - 0.026\ nA$, with a tolerance of $\pm 0.005 nA$. This range of input current clamps the firing rate between $2.63 - 3.55$ Hz. When the input current goes beyond the tolerance level, we increase that area's local feedback inhibitory weight (J_i) by a small value (Δ). Analytically, when $I_i^E - \frac{b_E}{a_E} > -0.026$, we increase or upregulate the corresponding local feedback inhibition, $J_i = J_i + \Delta$ of the area i; otherwise we downregulate the corresponding inhibitory weights $J_i = J_i - \Delta$. The process is repeated until all regions' firing rates reach a critical firing rate regime ($2.63 - 3.55$ Hz). We simulated the model with the FIC algorithm at different time windows of 10 *sec*.

Virtual focal lesion

The virtual focal lesion is introduced into an individual subject's structural connectome or anatomical topology by the targeted removal of a single node. Specifically, all connections to and from the focal lesioned site have been set to zero in the SC matrix. Thus, a lesioned center is isolated from its neighbors and becomes functionally non-interactive with the remaining intact network. The anatomical topology of the remaining network is kept invariant, except the lesioned node is functionally isolated. The lesioned site will not participate in or influence the functions (or dynamics) of the remaining intact network. A single node is removed to project the exclusive impact of a specific lesioned site. The rest of the remaining network is used in the simulation. Here we consider that the lesion only involves the deletion of a node ('gray matter ') and its afferent connections. In contrast, we do not attempt to model 'while-matter' volume, e.g., including lesions of 'fibers of passage' [Alstott et al., 2009]. We consider all 68 regions as lesion centers covering the whole cerebral cortex.

Virtual lesion model

When the DMF model is put on top of the virtually lesioned SC, we labeled them as the virtual lesion model. Individual node dynamics are governed by the stochastic DMF model spatially coupled via lesioned SC matrix. In principle, any other form of lesioned SC (real, virtual), if incorporated into the dynamical model, can produce similar effects of lesion depending on the lesion type, location, and extent.

Table 2: List of all 34 ROIs in each hemisphere. ROI ID represents the order of ROIs in the structural and functional connectivity matrices for each hemisphere.

Area index	Abbreviation	Full form
1	BSTS	Banks Of Superior Temporal Sulcus
2	CAC	Caudal Anterior Cingulate
3	CMF	Caudal Middle Frontal
4	CUN	Cuneus
5	ENT	Entorhinal
6	FITS	Fusiform
7	IP	Inferior Parietal
8	IT	Inferior Temporal
9	ISTH	Isthmus Cingulate
10	LOCC	Lateral Occipital
11	LOF	Lateral Orbito Frontal
12	LING	Lingual
13	MOF	Medial Orbito Frontal
14	MT	Middle Temporal
15	PARH	Parahippocampal
16	PARC	Paracentral
17	POPE	Pars Opercularis
18	PORB	Pars Orbitalis
19	PTRI	Pars Triangularis
20	PCAL	Pericalcarine
21	PCNT	Post Central
22	PC	Posterior Cingulate
23	PREC	Precentral
24	PCUN	Precuneus
25	RAC	Rostral Anterior Cingulate
26	RMF	Rostral Middle Frontal
27	SF	Superior Frontal
28	SF	Superior Parietal
29	ST	Superior Temporal
30	SMAR	Supra Marginal
31	FP	Frontal Pole
32	TP	Temporal Pole
33	TT	Transverse Temporal
34	INS	Insula

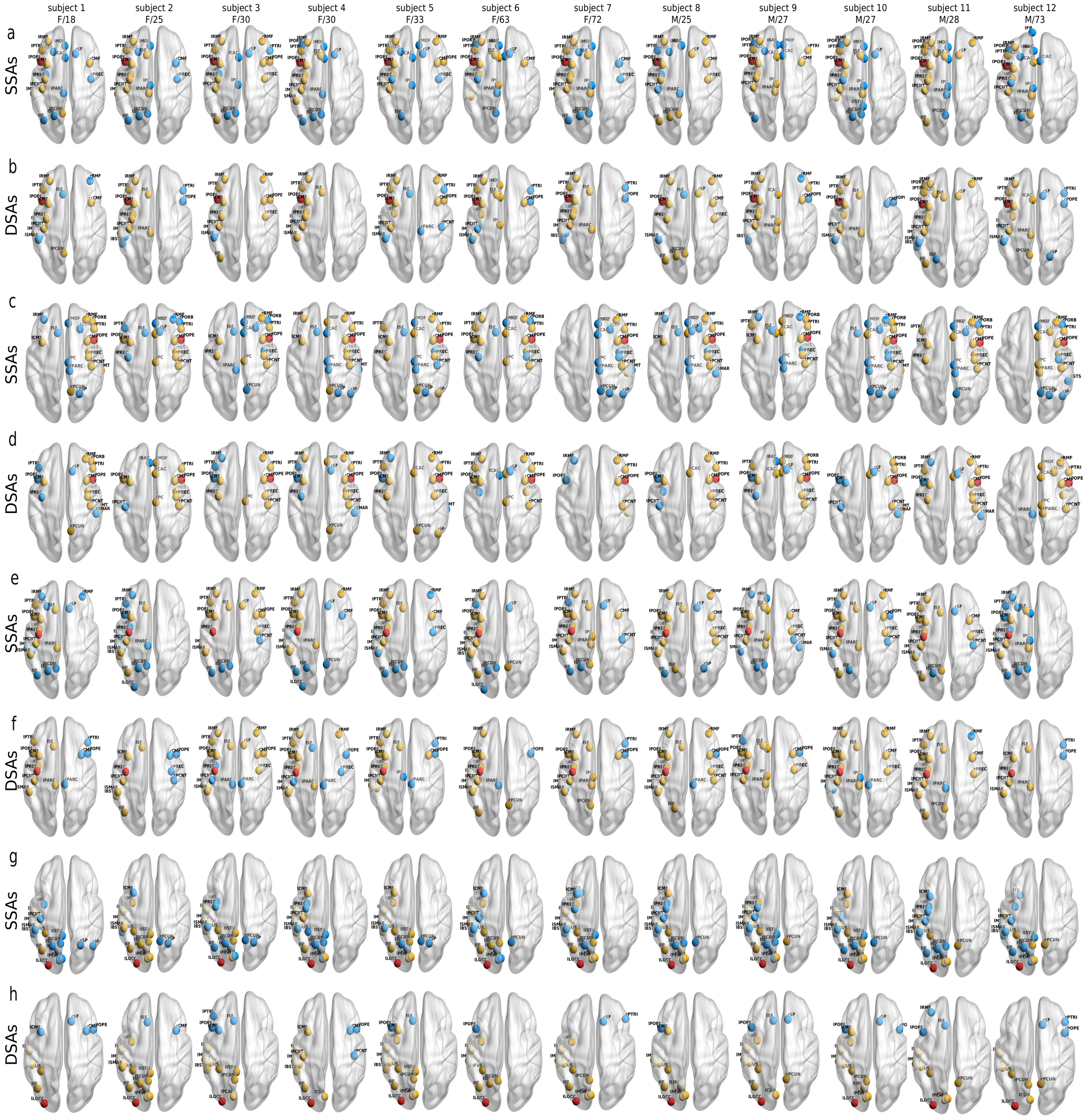

Figure 1: SSAs and DSAs for ten individual subjects corresponding to lesion centers at (a-b) lPOPE, (c-d) rPOPE, (e-f) lPREC, and (g-h) lLOCC. Red sphere represents lesion location. Common areas found in SSAs and DSAs, are shown in yellow, and unmatched areas in blue. Subjects' gender/age are written in the top of each brain.

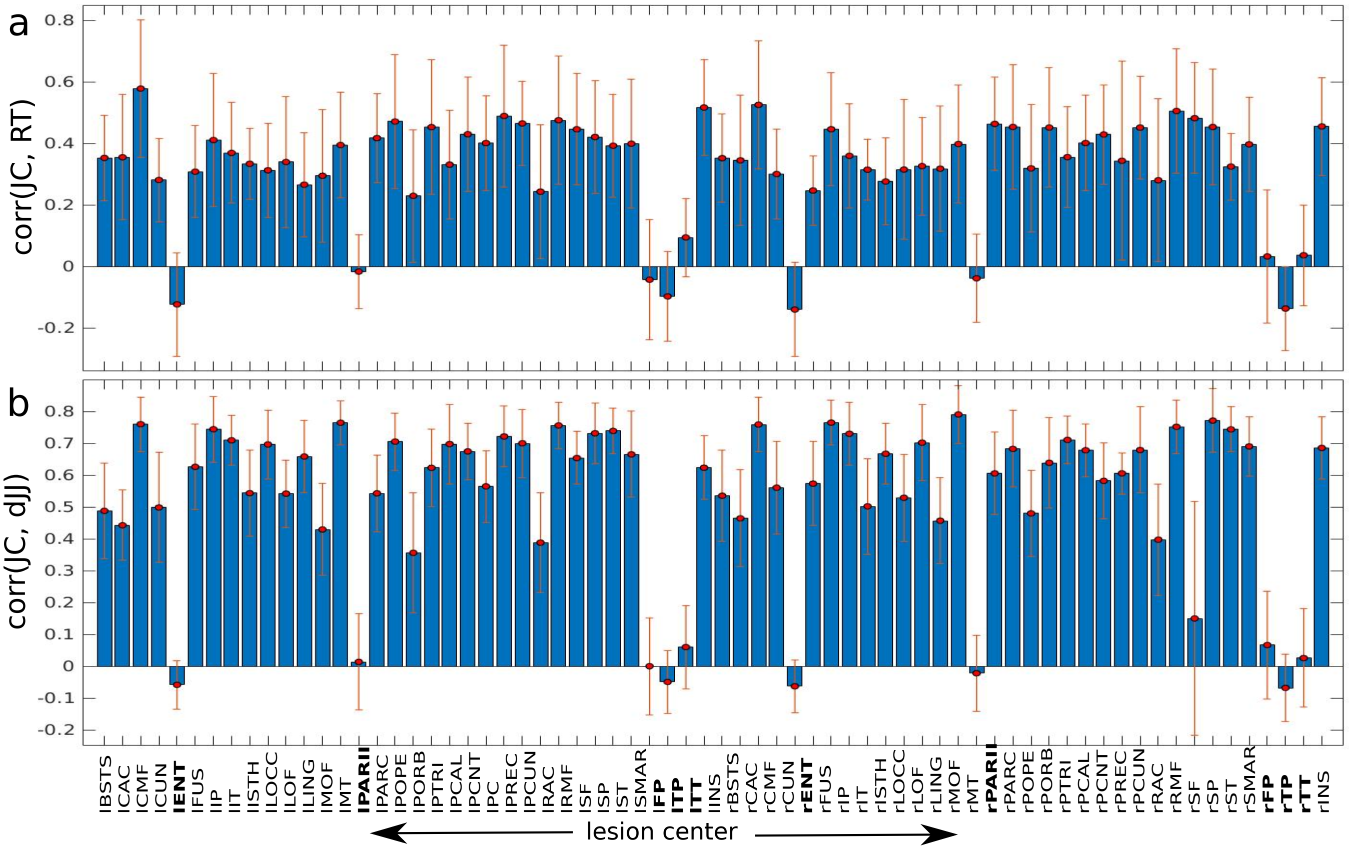

Figure 2: **Association between structural and dynamical measures for different lesion centers and subjects.** (a) Correlation between JC and RT has been determined over all the subjects for different lesion centers. The lesion center at a higher degree area displays a positive correlation, except the regions with lower connections show less or negative correlations. Blue bars are obtained by averaging the correlation over the subjects. Subject-wise variations are depicted by red error bars. (b) Similar trends in correlations between JC and dJJ are observed. Lesion centers are listed below the figure. The areas with low or negative correlations are marked in bold.

Details of the regions with lower connectivity

We also investigate the anatomical biases of the nodes with low and negative correlations. The distribution of dJJ for a single subject at the lesion center, lENT, is shown in SI Appendix, Fig. S3c. The nodes do show very small changes due to lesions at lENT. We also looked at the anatomical connections of the lENT (SI Appendix, Fig. S3d). The brain area lENT is sparsely connected to the rest of the network. We further analyzed all the areas' average anatomical strength and degree distributions (SI Appendix, Figs. S3e,f).

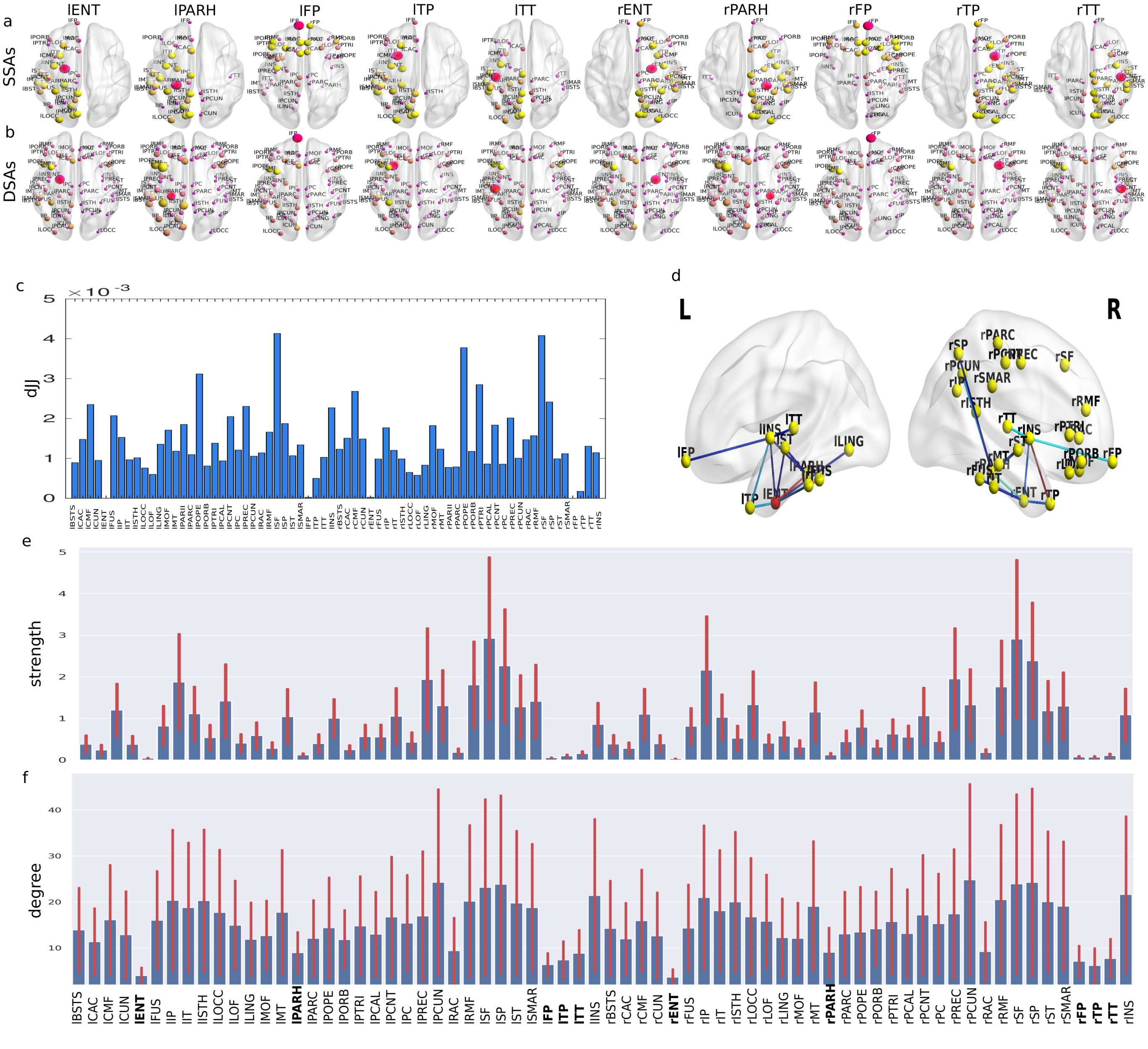

Figure 3: The regions written in bold in Fig. S2 are analyzed at the group level. Findings of SSAs and DSAs for a few lesion sites are given in (a) and (b), respectively. Lesion centers are written on the top of each glass brain plot. Lesion at lENT is an example for a single subject, depicting the distribution of dJJ across all areas in (c), and anatomical connections of the lENT in (d). (e) Average anatomical strength and (f) degree of all the areas. Regions with lower connectivities and node weights are marked in bold.

Group level analysis of SSAs and DSAs

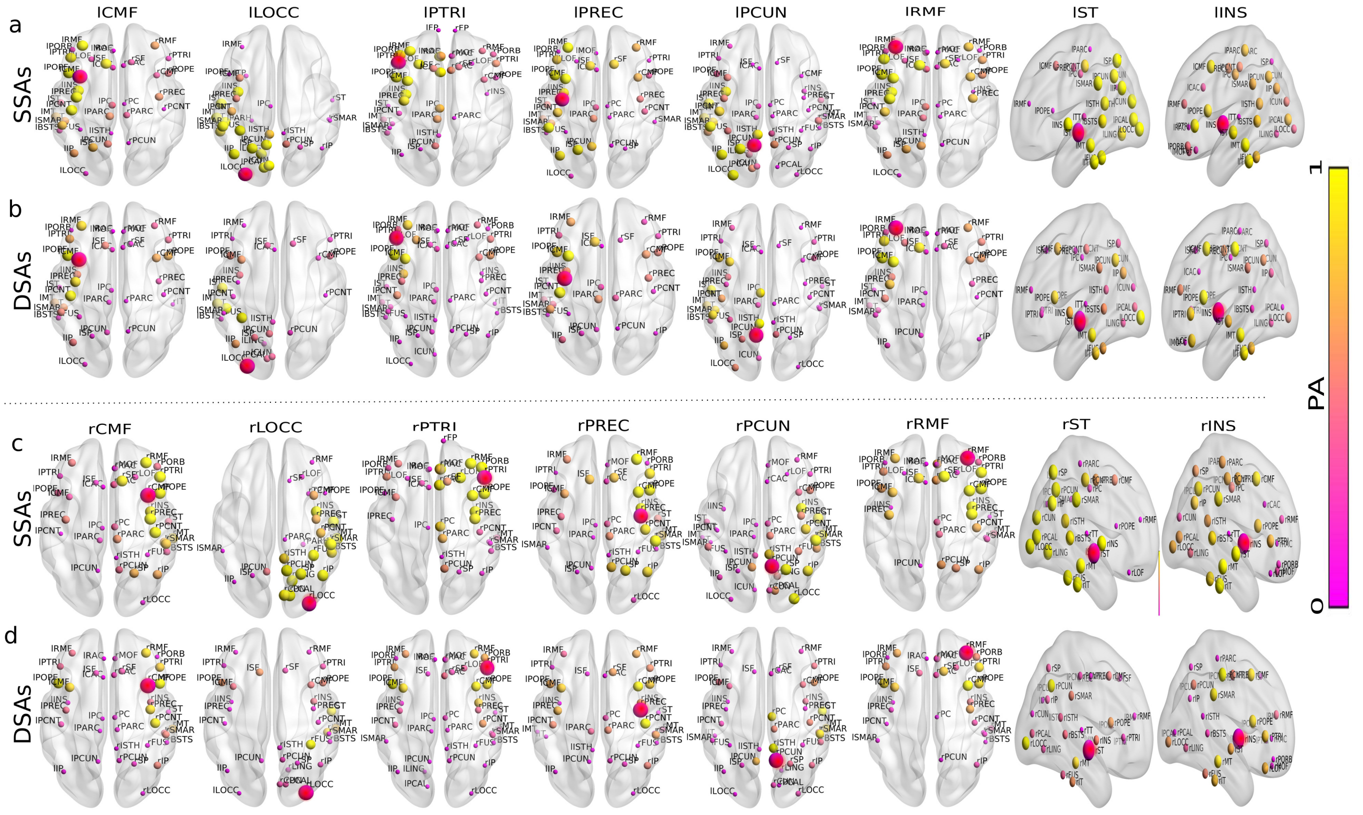

Figure 4: Group level analysis. The identified SSAs and DSAs corresponding to different lesion centers are shown in (a,c) and (b,d), respectively. Abbreviations of lesion sites are written on the top of each brain plot. The red sphere is the lesion site. Yellow areas have higher PA, as they are in almost all subjects. Areas in pink with lower PA indicate that those areas are less probable to be the potential compensatory candidates in all subjects.

ROI-wise FC analysis over all subjects for different lesion centers

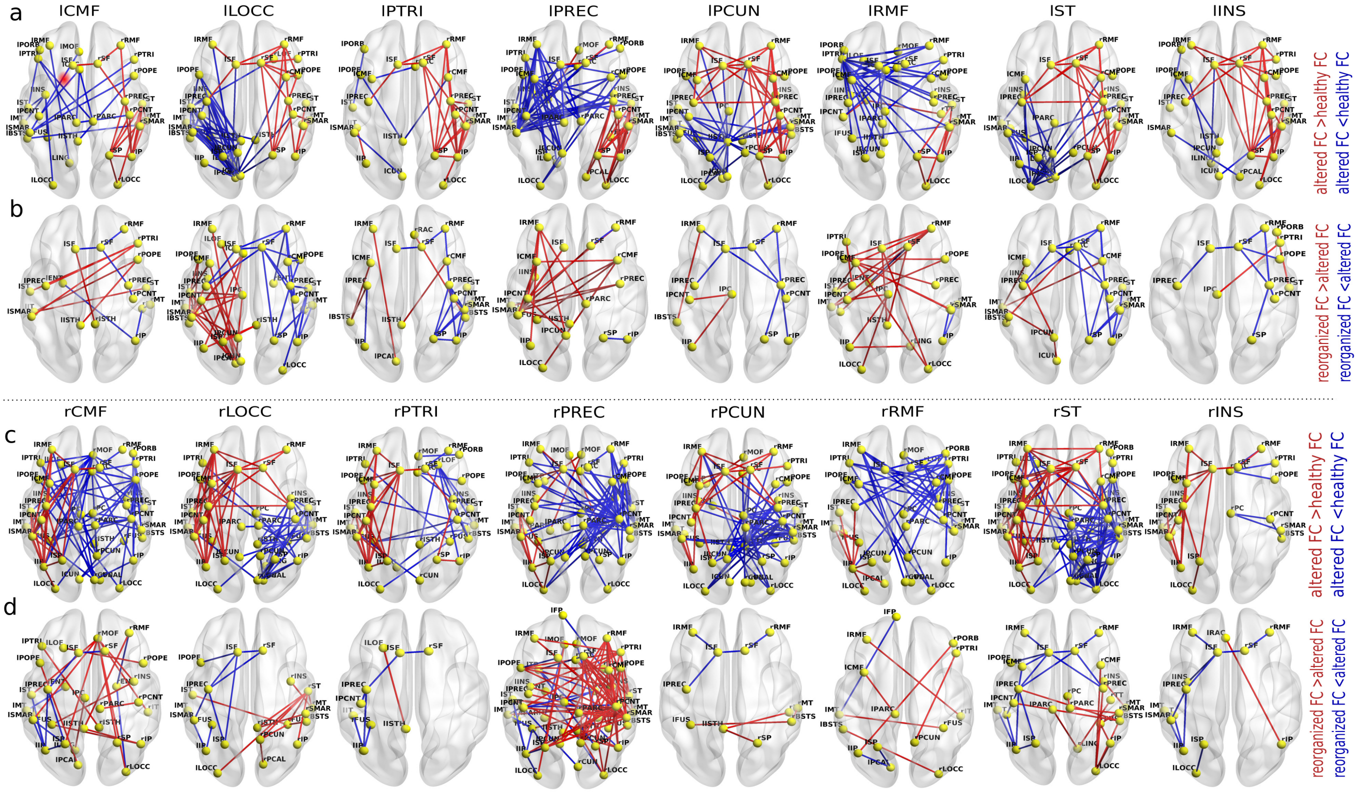

Figure 5: ROI-wise FC analysis for different lesion centers. Significantly changed and rewired links with associated regions are shown in (a,c) and (b,d), respectively. Lesion centers abbreviations are written on the top of each brain. Red and blue edges represent significant increase and decrease in cohesion, respectively.

[Seghier et al., 2005, Guzzetta et al., 2010]

Table 3: Lesion centers, SSAs, DSAs and correlation between JC and RT. We choose only those areas as SSAs and DSAs, which have $PA > 0.8$.

Lesion center (area index)	SSAs	DSAs	Correlation JC and RT Mean(SD)	Correlation JC and dJJ Mean(SD)	
lCAC (2)	lRAC, lPOPE, lPTRI, lPORB, lLOF, lPC, lPARC, lISTH, lINS, rCAC	lPOPE, lCMF, lPC, lPTRI, lISTH, lPCUN, rCMF, rPOPE, lRMF, lPCNT	0.36(0.20)	0.44(0.11)	
lCMF (3)	lRMF, lPTRI, lPOPE,lSF, lINS, lREC, lPCNT,lMT	lPOPE, lPCNT, lPREC, lRMF, rPOPE, lMT, lPTRI, rCMF, lIT, lINS, lSF	0.58(0.22)	0.76(0.08)	
lCUN (4)	lFUS, lISTH, lLING, lMT, lPCAL, lST, lTT, lINS, lIT, lPCUN, lLOCC, lPC	lPCAL, lFUS, lISTH, lPOPE, lPCUN, lCMF, lLOCC, rPOPE, lIT, lMT	0.28(0.14)	0.50(0.17)	
lENT (5)	lISTH, lLING, lPARH, lPCAL, lTP, lCUN, lLOCC, lINS, lFUS, lIT	lCMF, lFUS, lPOPE, lCAC, lCUN, lBSTS, lIT, lIP, lISTH, rPOPE	-0.12(0.17)	-0.06(0.08)	
lIP (7)	lIT, lINS, lPREC, lST, lPCNT, lMT, lLOCC, lSMAR,lFUS,lSP, lPCUN	lCMF, lIT, lMT, lPCUN, lSP, lST, lSMAR, lPCNT, lLOCC, lPOPE, lPREC	0.41(0.22)	0.74(0.10)	
lIT (8)	lBSTS, lFUS, lIP, lLOCC, lMT lSP, lST, lSMAR, lINS, lLING, lPCAL, lPCUN	lMT, lFUS, lCMF, lLOCC, lPOPE, lIP, lPCNT, lPCUN, lST, lSMAR, lPREC	0.37(0.16)	0.71(0.08)	
lLOCC (10)	lPCAL, lCUN, lIP, lLING, lPCUN, lSP, lFUS, lSMAR, lMT, lST, lINS	lIT, lFUS, lST, lMT, lIP, lCMF, lPOPE, lSP, lINS, lPCUN	0.31(0.15)	0.70(0.11)	
lPARH (15)	lCUN, lISTH, lPCAL, lTP, lTT, lINS, lLING, lFUS, lP,C lST	lISTH, lCMF, lFUS, lPOPE, lIT, lLING, lLOCC, lBSTS, rPOPE, lCAC lMT	-0.02 (0.12)	0.01 (0.15)	
lPOPE (17)	lCMF, lPTRI, lPREC, lRMF, lINS, lPCNT, lPCUN, lPC, lCAC, rSF, lPARC, lLOF	lCMF, lPTRI, lPCNT lRMF, lPREC, lSF, lINS, lSMAR, rCMF, lIT	0.47(0.22)	0.71(0.09)	[Winhuisen et al., 200
lPORB (18)	lPOPE, lPTRI, lLOF, lRAC, lCMF, lINS, rCAC, rRAC, rMOF	lCMF, lLOF, lPOPE, lPTRI, rPOPE, rPTRI, lRMF, lINS, rCMF, lPCNT	0.23(0.22)	0.36(0.19)	
lPTRI (19)	lPORB, lINS, lCMF, lLOF, lMOF, lPOPE, lRMF, lCAC, lPREC, lPC, lRAC, lPARC	lPOPE, lCMF, lRMF, lINS, lLOF, rPOPE, lPCNT, rCMF, rPTRI, lPREC, rRMF, lPORB	0.45(0.22)	0.62(0.12)	
lPCNT (21)	lCMF, lSP, lSMAR, lINS, lIP, lPREC, lST, lMT, lPCUN, lPARC, lST	lCMF, lPOPE, lPREC, lSMAR, lINS, lPARC, lIT, rPOPE, lMT, lIP, rPCNT	0.43(0.19)	0.67(0.09)	
lPREC (23)	lRMF, lCMF, lPOPE, lSMAR, lSP, lIP, lSF, rSF, lPCUN, lST, lPCNT, lINS	lCMF, lPCNT, lPOPE, lSF, lRMF, lSMAR, rCMF, lPARC, lINS, rPOPE, rPREC	0.49(0.23)	0.72(0.09)	
lPCUN (24)	lLOCC, lSP, lIP, lSMAR, lST, lINS, lISTH, lMT, lPCNT, lIT, lBSTS, rPCUN	lISTH, lST, lPC, lSMAR, lPOPE, lCMF, lIT, lMT, lIP, lPCNT, lLOCC	0.47(0.14)	0.70(0.11)	
lRMF (26)	lCMF, lPOPE, lPTRI, lPREC, lSF, lINS, rSF, lPCNT, rRMF, rCMF, rPREC, lSP	lCMF, lPOPE, lPTRI, lSF, lPREC, rRMF, rPOPE, rCMF, lPCNT, lINS, rPTRI	0.48(0.21)	0.76(0.07)	
lSF (27)	lCMF, lPCNT, lRMF, lPREC, lPTRI, lPOPE, lSMAR, rPREC, rRMF, rSF	lRMF, lPOPE, lPREC, lCMF, lPTRI, lPCNT, lINS, rCMF, rRMF, rSF, rPREC	0.45(0.18)	0.65(0.08)	
lSP (28)	lIP, lIT, lLOCC, lMT, lPCNT, lPCUN, lST, lSMAR, lPREC, lINS	lIP, lSMAR, lPCUN, lCMF, lPCNT, lST, lIT, lPREC, lMT, lLOCC, lINS	0.42(0.18)	0.73 (0.10)	
lST (29)	lCUN, lFUS, lIP, lIT, lLOCC, lMT, lSP, lSMAR, lINS, lPCAL	lLOCC, lMT, lPOPE, lPCUN, lIT, lCMF, lIP, lINS, lFUS, lBSTS, lSMAR	0.39(0.17)	0.74(0.07)	
lSMAR (30)	lIP, lSP, lST, lINS, lMT, lPCNT, lPCUN, lLOCC, lIT, lPREC, lCMF, lISTH	lPCNT, lCMF, lPOPE, lMT, lSP, lPREC, lPCUN, lINS, lIT, lBSTS, lST, lIP	0.40(0.21)	0.67(0.14)	
lFP (31)	lCAC, lLOF, lMOF, lPORB, lPTRI, lRAC, lINS, rRAC, rCAC, rFP, rMOF, rPORB	lCMF, lPOPE, lCAC, lBSTS, lCUN, lENT, lFUS, rPOPE, lIT, lLOF, lISTH	- 0.04(0.19)	0.00 (0.15)	
lTP (32)	lBSTS, lENT, lLOCC, lLING, lPARH, lPCAL, lMT, lLOF, lIT, lTT, lINS	lCMF, lFUS, lIT, lPOPE, lBSTS, lMT, lPTRI, lCAC, lLOCC, lINS, lISTH	-0.10 (0.15)	-0.05 (0.10)	
lTT (33)	lBSTS, lCUN, lISTH, lINS, lMT, lPCAL, lST, lPC, lPCUN, lSMAR	lPOPE, lCMF	0.09 (0.13)	0.06 (0.13)	
lINS (34)	lMT, lIT, lPTRI, lST, lCMF, lPOPE, lBSTS, lPARC, lFUS, lPCAL, lPCUN, lISTH	lPCNT, lMT, lPOPE, lCMF, lIT, lLOF, lPTRI, lFUS, lST, lPREC, lPCUN	0.52(0.16)	0.62(0.10)	

Table 3 (Cont.). Lesion centers, SSAs, DSAs and correlation between JC and RT. We choose only those areas as SSAs and DSAs, which have $PA > 0.8$.

Lesion center (area index)	SSAs	DSAs	Correlation JC and RT Mean(SD)	Correlation JC and dJJ Mean(SD)	
rCAC (36)	rMOF, rRAC, rPORB, rPTRI, rPC, rPARC, rPOPE, lCAC, rLOF, rINS, rCMF, lPORB	lPOPE, lCMF, rPOPE, rPC, rCMF, rPTRI, lRMF, lPTRI, rRMF, lPCNT	0.36(0.21)	0.46(0.15)	
rCMF (37)	rPTRI, rPREC, rPOPE, rPCNT, rRMF, rSF, rINS, rMT, rSMAR, rIT, rSP	rPOPE, lPOPE, rPCNT, rPREC, rRMF, lCMF, rPTRI, rMT, rIP, rSMAR, lPREC, lRMF	0.58(0.21)	0.76(0.08)	
rENT (39)	rCUN, rLING, rPARH, rPCAL, rTP, rISTH, rLOCC, rLOF, rTT, rFUS	lCMF, lCAC, lFUS, lCUN, lPOPE, lIT, rBSTS lENT, rIP lISTH	-0.14(0.15)	-0.10(0.08)	
rIP (41)	rLOCC, rMT, rPCUN, rSP, rST, rSMAR, rINS, rIT, rPCNT, rFUS, rPREC	rSP, rCMF, rMT, rSMAR, rIT, rPCNT, rST, rBSTS, rLOCC, rPOPE, rPREC	0.41(0.18)	0.76(0.07)	
rIT (42)	rFUS, rLOCC, rLING, rMT, rPCAL, rSP, rST, rSMAR, rINS, rIP, rBSTS, rPCUN	rFUS, rMT, rCMF, rLOCC, lPOPE, rPOPE, rST, lCMF, rIP, rPCNT	0.37(0.17)	0.73(0.10)	
rLOCC (44)	rFUS, rIP, rIT, rLING, rMT, rPCAL, rPCUN, rSP, rST, rSMAR, rINS, rCUN	rFUS, rST, rIT, rMT, lPOPE, lSF, rPOPE, rCMF, lCMF, rINS, rCUN	0.31(0.14)	0.67(0.10)	
rPOPE (51)	rCMF, rPTRI, rPREC, rRMF, rINS, rCAC, rPCNT, rPC, rMOF, rPARC, rPCUN, rPORB	lPOPE, rCMF, rPTRI, lCMF, rRMF, rPCNT, rSF, lPTRI, rPREC, lRMF, rSMAR, rCAC	0.47(0.20)	0.68(0.12)	
rPORB (52)	rCAC, rLOF, rMOF, rPTRI, rRAC, rINS, rCMF, rPOPE, rFP, rPC	lPOPE, rPTRI, rPOPE, lCMF, rCMF, rLOF, lPTRI, rRMF, rMOF, rINS	0.23(0.21)	0.48(0.14)	
rPTRI (53)	rCMF, rLOF, rMOF, rPOPE, rPORB, rRMF, rINS, rPREC, rCAC, rPC	lPOPE, rCMF, rPOPE, lCMF, rRMF, rINS, rPORB, lPTRI, lRMF, rPCNT, rCAC	0.45(0.19)	0.64(0.14)	
rPCNT (55)	rCMF, rPREC, rSMAR, rINS, rSP, rIP, rPCUN, rMT, rST, rPARC	rPREC, rSMAR, rCMF, rPOPE, rINS, lPCNT, lPOPE, rPARC, lCMF, rPTRI	0.43(0.16)	0.68(0.08)	
rPREC (57)	rCMF, rRMF, rSMAR, rPOPE, rINS, rPTRI, rPCNT, rSP, rIP, rPCUN, rMT, rSF	rPCNT, rCMF, lCMF, rPOPE, lPREC, lPOPE, rRMF, rSF, rSMAR, rINS, rPTRI, lPCNT	0.49(0.32)	0.61(0.06)	[Connolly and et al.,
rPCUN (58)	rLOCC, rSP, rSMAR, rINS, rST, rMT, rIP, rPCNT,rISTH,rIT,lPCUN,rBSTS	rISTH, rST, rPC, rSMAR, rMT, lPCUN, rPOPE, rPARC, rPCNT, rIT	0.47(0.17)	0.68(0.14)	
rRMF (60)	rCMF, rPOPE, rPREC, rSF, rINS, rPTRI, lSF, lCMF, lPREC, lRMF	rCMF, lPOPE, rPOPE, rPTRI, lCMF, rSF, lRMF, rPREC, lPTRI, rPORB	0.48(0.20)	0.75(0.08)	
rSF (61)	rCMF, rPTRI, rPREC, rRMF, rPCNT, rIP, lPREC, lRMF, lPOPE, lSF	rRMF, rPOPE, lPOPE, rPREC, lCMF, rCMF, rPTRI, lRMF, lPREC, lSF, rCAC	0.4(0.18)5	0.20(0.37)	
rSP (62)	rIP, rIT, rMT, rPCNT, rPCUN, rST, rSMAR, rINS, rLOCC, rPREC, rBSTS	rSMAR, rIP, rPCUN, lPCUN, rPCNT, rST, rMT, rLOCC, rPREC, rINS	0.42(0.19)	0.77(0.10)	
rST (63)	rFUS, rIP, rIT, rLOCC, rMT, rPCAL, rSMAR, rINS, rCUN, rLING, rPCUN	rLOCC, rPCUN, rMT, rPOPE, rIT, rSMAR, rINS, rCMF, rBSTS, lPOPE	0.39(0.11)	0.74(0.07)	
rSMAR (64)	rIP, rMT, rPCNT, rSP, rST, rINS, rPREC, rPCUN, rIT, rLOCC, rCMF, rBSTS	rPCNT, rPOPE, rPCUN, rSP, rINS, rCMF, rST, rPREC, rMT, rBSTS, rPTRI	0.40(0.15)	0.69(0.09)	
rFP (65)	rCAC, rLOF, rMOF, rPORB, rPTRI, rRAC, lRAC, rINS , rTP , lMOF	lCMF, lPOPE, lCAC	0.03 (0.22)	0.10 (0.10)	
rTP(66)	rCUN, rENT, rLOCC , rLOF, rLING, rPARH , rPCAL , rTT, rBSTS	lCMF, lPOPE	-0.14 (0.14)	-0.07(0.15)	
rTT(67)	rBSTS, rCUN, rISTH, rINS, rPCAL, rPC, rST, rPARH, rPCUN, rSMAR, rMT	lCMF, lPOPE	0.04 (0.16)	0.02(0.15)	
rINS (68)	rIT, rMT, rST, rSMAR, rCMF, rPCUN, rBSTS, rFUS, rIP, rPOPE, rPTRI, rLOCC	rPCNT, rMT, rSMAR, rCMF, rPOPE, rPTRI, rIT, rLOF, rST, rLOCC, lPOPE, rFUS	0.52(0.16)	0.69(0.10)	

Correlation between JC and other properties of SC

If the nearest neighbours of a node are also directly connected to each other they form a cluster. The clustering coefficient quantifies the number of connections that exist between the nearest neighbours of a node as a proportion of the maximum number of possible connections18

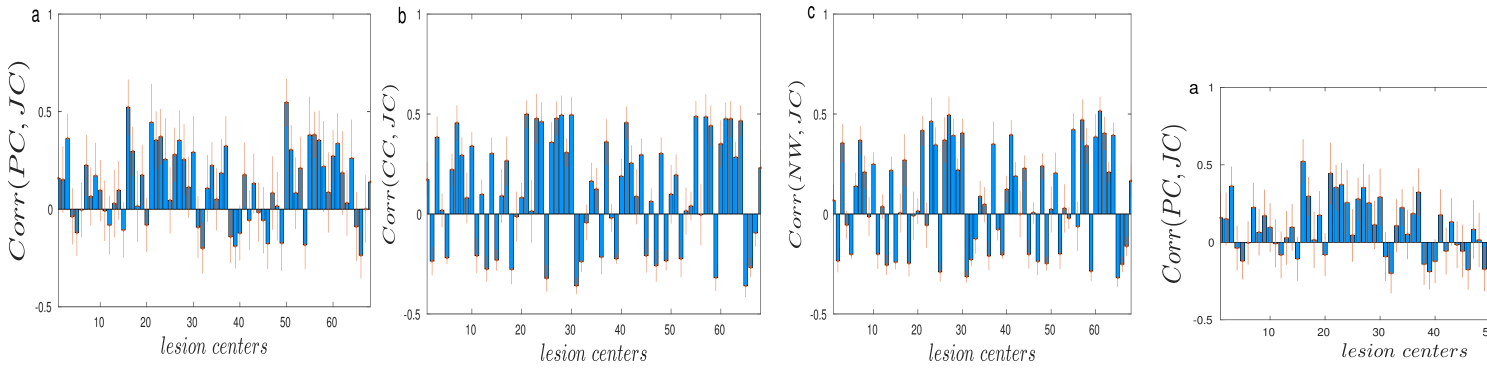

Figure 6: **Correlation between JC and other network measures.** Mean correlation values are obtained averaging over subjects for different lesion centers. No specific or conclusive patterns are observed in the correlations between JC-PC, JC-CC and JC, NW. PC, CC and NW are participation coefficient, clustering coefficient and node weight. All the network measures are obtained using BCT tools box.

Functional network properties under three given conditions

The alteration in functional brain network due to structural damage as an immediate impact of lesion and FC re-organization after restoring E-I balance are reconfirmed by measuring several network properties, such as transitivity, average characteristic path length, modularity, and global efficiency, presented in Fig. 7. The network properties computed from healthy, altered, and re-organized FCs are shown in yellow, red, and blue bars, respectively, see Figs. 7(a-d). Transitivity, average characteristic path length, and modularity significantly increased in the altered FCs, when they deviated from the healthy condition due to lost E-I homeostasis. However, the FCs regain near-healthy conditions when the homeostatic balance is re-established after the lesion. The three network properties are significantly decreased in the re-organized FCs compared to the altered FCs. Conversely, global efficiency significantly decreased ($p < 0.001$) in the altered FC and increased after the re-organization. Alteration/re-organization is further reconfirmed by the probability distributions of FC weights from the three conditions. Distributions for healthy, altered, and re-organized FCs of a single subject are shown in purple, red, and green in Fig. 7e. Blue, red, and green lines indicate the mean values of the three distributions, respectively.

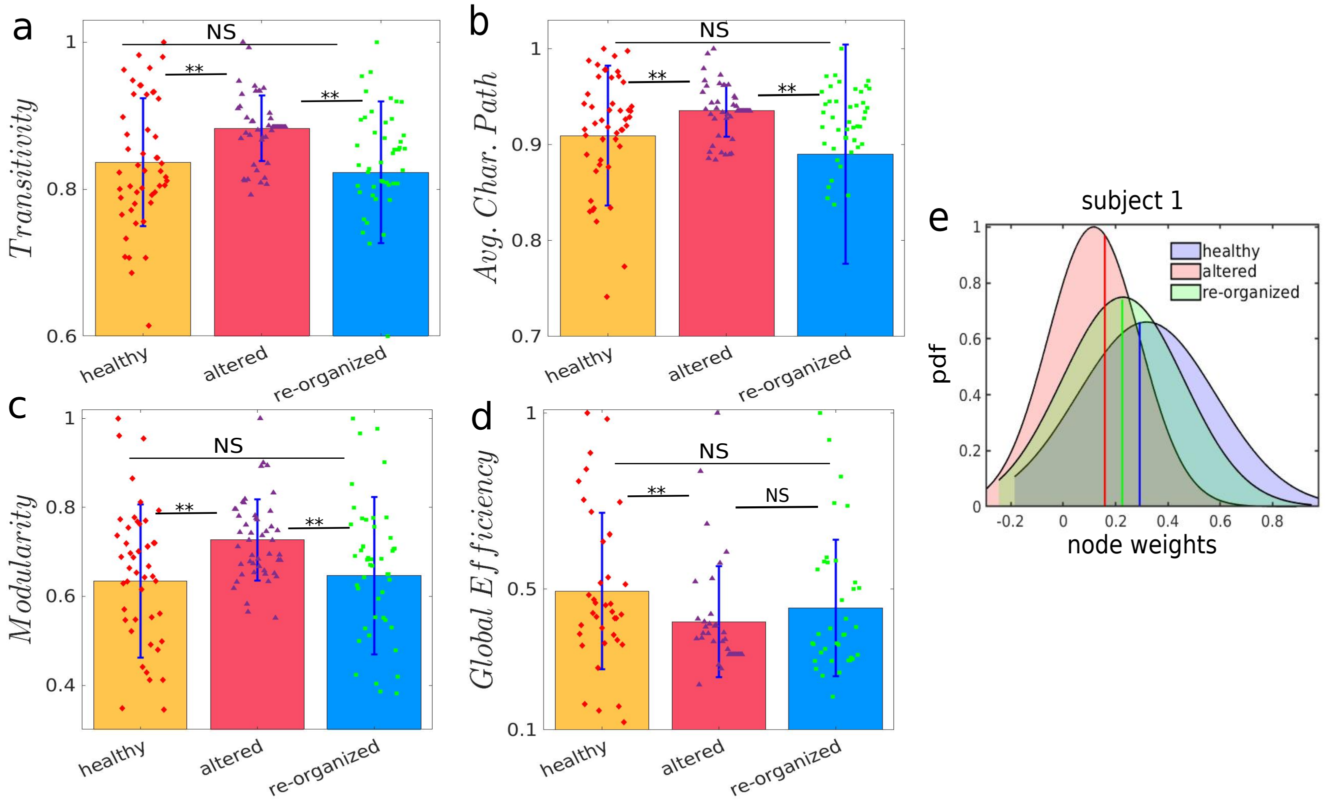

Figure 7: **Functional network properties.** (a) Transitivity, (b) average characteristic path length, (c) modularity, and (d) global efficiency derived from healthy, altered, and re-organized FCs are plotted in yellow, red, and blue bars, respectively. Deviation over the subjects is shown by the error bar. (e) Probability distributions of the three FCs, driven from the three conditions, confirm FC reshaping after lesion. $**p < 0.001$, 'NS' for not significant. Network measures are obtained using BCT tools box.

Definition of network properties

(i) Transitivity [Humphries and Gurney, 2008], or clustering coefficient, measures the tendency of the nodes to cluster together. High transitivity means that the network contains communities or groups of nodes that

are densely connected internally. Transitivity of a graph with degree sequence k is $CC = \frac{1}{n}\frac{[\langle k^2\rangle - \langle k\rangle]^2}{\langle k\rangle^3}$, where $\langle k\rangle = 1/n\sum_i k_i$ is the mean degree and $\langle k^2\rangle = 1/n\sum_i k_i^2$ is the mean square degree.

(ii) Characteristic path length [Watts and Strogatz, 1998], $L = \frac{1}{n}\sum_{i\in N}\frac{\sum_{j\in N, j\neq i} d_{ij}}{n-1}$, where N is the set of all nodes; n is the total nodes; d_{ij} is the weighted shortest path length between i and j. The characteristic path length for weighted graphs is an estimate of proximity. The global efficiency is the average of the inverse shortest path length and is inversely related to the characteristic path length. The local efficiency is the global efficiency computed on the node's neighborhood and is related to the clustering coefficient.

(iii) Modularity [Newman, 2006], $M = \frac{1}{l}\sum_{(i,j\in N)}\left[w_{ij} - \frac{k_i k_j}{l}\right]\delta_{m_i m_j}$, where l is the sum of weights in the network; w_{ij} is the connectivity weight between nodes i and j; k_i, k_j are the weighted degrees of nodes i and j, respectively. Modularity gives network resilience and adaptability, measuring the degree of segregation. Communities are subgroups of densely interconnected nodes sparsely connected with the rest of the network. In the case of a functional network, modularity signifies coherent clusters of functional modules.

(iv) Global efficiency [Latora and Marchiori, 2001], $E = \frac{1}{n}\sum_{i\in N}\frac{\sum_{j\in N, j\neq i} d_{ij}^{-1}}{n-1}$, where N is the set of all nodes; n is the total number of nodes; d_{ij} is the weighted shortest path length between node i and j. It captures the integration property of a network.

References

[Alstott et al., 2009] Alstott, J., Breakspear, M., Hagmann, P., Cammoun, L., and Sporns, O. (2009). Modeling the impact of lesions in the human brain. *PLoS computational biology*, 5(6):e1000408.

[Breier et al., 2009] Breier, J. I., Juranek, J., Maher, L. M., Schmadeke, S., Men, D., and Papanicolaou, A. C. (2009). Behavioral and neurophysiologic response to therapy for chronic aphasia. *Archives of Physical Medicine and Rehabilitation*, 90(12):2026–2033.

[Burns and Webb, 1976] Burns, B. D. and Webb, A. (1976). The spontaneous activity of neurones in the cat's cerebral cortex. *Proc of the Roy Soc London. Series B. Bio Sci*, 194(1115):211–223.

[Cao et al., 1999] Cao, Y., Vikingstad, E. M., George, K. P., Johnson, A. F., and Welch, K. (1999). Cortical language activation in stroke patients recovering from aphasia with functional mri. *Stroke*, 30(11):2331–2340.

[Chivukula et al., 2018] Chivukula, S., Pikul, B. K., Black, K. L., Pouratian, N., and Bookheimer, S. Y. (2018). Contralateral functional reorganization of the speech supplementary motor area following neurosurgical tumor resection. *Brain and language*, 183:41–46.

[Connolly and et al., 1994] Connolly, A. M. and et al. (1994). Course and outcome of acute cerebellar ataxia. *Ann of Neur*, 35(6):673–679.

[Deco and Jirsa, 2012] Deco, G. and Jirsa, V. K. (2012). Ongoing cortical activity at rest: criticality, multistability, and ghost attractors. *Jour of Neurosci*, 32(10):3366–3375.

[Deco et al., 2014] Deco, G., Ponce-Alvarez, A., Hagmann, P., Romani, G. L., Mantini, D., and Corbetta, M. (2014). How local excitation–inhibition ratio impacts the whole brain dynamics. *Jour of Neurosci*, 34(23):7886–7898.

[Desikan et al., 2006] Desikan, R. S., Ségonne, F., Fischl, B., Quinn, B. T., Dickerson, B. C., Blacker, D., Buckner, R. L., Dale, A. M., Maguire, R. P., Hyman, B. T., et al. (2006). An automated labeling system for subdividing the human cerebral cortex on mri scans into gyral based regions of interest. *Neuroimage*, 31(3):968–980.

[Guzzetta et al., 2010] Guzzetta, A., D'ACUNTO, G., Rose, S., Tinelli, F., Boyd, R., and Cioni, G. (2010). Plasticity of the visual system after early brain damage. *Developmental Medicine & Child Neurology*, 52(10):891–900.

[Humphries and Gurney, 2008] Humphries, M. and Gurney, K. (2008). Network 'small-world-ness': a quantitative method for determining canonical network equivalence. *PloS One*, 3(4):e0002051.

[Johansen-Berg and et al., 2002] Johansen-Berg, H. and et al. (2002). The role of ipsilateral premotor cortex in hand movement after stroke. *Proc Natl Acad Sci*, 99(22):14518–14523.

[Langhorne et al., 2009] Langhorne, P., Coupar, F., and Pollock, A. (2009). Motor recovery after stroke: a systematic review. *The Lancet Neurology*, 8(8):741–754.

[Latora and Marchiori, 2001] Latora, V. and Marchiori, M. (2001). Efficient behavior of small-world networks. *Phys. Rev. Lett.*, 87(19):198701.

[Newman, 2006] Newman, M. E. (2006). Modularity and community structure in networks. *Proc Nat Acad Sci*, 103(23):8577–8582.

[Schirner and et al., 2015] Schirner, M. and et al. (2015). An automated pipeline for constructing personalized virtual brains from multimodal neuroimaging data. *NeuroImage*, 117:343–357.

[Seghier et al., 2005] Seghier, M. L., Lazeyras, F., Zimine, S., Saudan-Frei, S., Safran, A. B., and Huppi, P. S. (2005). Visual recovery after perinatal stroke evidenced by functional and diffusion mri: case report. *BMC neurology*, 5(1):1–8.

[Shadlen and Newsome, 1998] Shadlen, M. N. and Newsome, W. T. (1998). The variable discharge of cortical neurons: implications for connectivity, computation, and information coding. *Jour of Neurosci*, 18(10):3870–3896.

[Sharp et al., 2010] Sharp, D. J., Turkheimer, F. E., Bose, S. K., Scott, S. K., and Wise, R. J. (2010). Increased frontoparietal integration after stroke and cognitive recovery. *Ann of Neur*, 68(5):753–756.

[Small and et al., 2002] Small, S. L. and et al. (2002). Cerebellar hemispheric activation ipsilateral to the paretic hand correlates with functional recovery after stroke. *Brain*, 125(7):1544–1557.

[Szaflarski and et al., 2013] Szaflarski, J. P. and et al. (2013). Recovered vs. not-recovered from post-stroke aphasia: the contributions from the dominant and non-dominant hemispheres. *Rest Neuro Neurosci*, 31(4):347–360.

[Turkeltaub et al., 2012] Turkeltaub, P. E., Coslett, H. B., Thomas, A. L., Faseyitan, O., Benson, J., Norise, C., and Hamilton, R. H. (2012). The right hemisphere is not unitary in its role in aphasia recovery. *Cortex*, 48(9):1179–1186.

[Ward et al., 2003] Ward, N., Brown, M., Thompson, A., and Frackowiak, R. (2003). Neural correlates of motor recovery after stroke: a longitudinal fmri study. *Brain*, 126(11):2476–2496.

[Watts and Strogatz, 1998] Watts, D. J. and Strogatz, S. H. (1998). Collective dynamics of 'small-world'networks. *Nature*, 393(6684):440–442.

[Winhuisen et al., 2005] Winhuisen, L., Thiel, A., Schumacher, B., Kessler, J., Rudolf, J., Haupt, W. F., and Heiss, W. D. (2005). Role of the contralateral inferior frontal gyrus in recovery of language function in poststroke aphasia: a combined repetitive transcranial magnetic stimulation and positron emission tomography study. *Stroke*, 36(8):1759–1763.

[Wong and Wang, 2006] Wong, K.-F. and Wang, X.-J. (2006). A recurrent network mechanism of time integration in perceptual decisions. *Jour of Neurosci*, 26(4):1314–1328.
